# Dynamic Gut Microbiome across Life History of the Malaria Mosquito *Anopheles gambiae* in Kenya

**DOI:** 10.1371/journal.pone.0024767

**Published:** 2011-09-21

**Authors:** Ying Wang, Thomas M. Gilbreath, Phanidhar Kukutla, Guiyun Yan, Jiannong Xu

**Affiliations:** 1 Biology Department, New Mexico State University, Las Cruces, New Mexico, United States of America; 2 Program in Public Health, University of California Irvine, Irvine, California, United States of America; French National Centre for Scientific Research - Université Aix-Marseille, France

## Abstract

The mosquito gut represents an ecosystem that accommodates a complex, intimately associated microbiome. It is increasingly clear that the gut microbiome influences a wide variety of host traits, such as fitness and immunity. Understanding the microbial community structure and its dynamics across mosquito life is a prerequisite for comprehending the symbiotic relationship between the mosquito and its gut microbial residents. Here we characterized gut bacterial communities across larvae, pupae and adults of *Anopheles gambiae* reared in semi-natural habitats in Kenya by pyrosequencing bacterial 16S rRNA fragments. Immatures and adults showed distinctive gut community structures. Photosynthetic *Cyanobacteria* were predominant in the larval and pupal guts while *Proteobacteria* and *Bacteroidetes* dominated the adult guts, with core taxa of *Enterobacteriaceae* and *Flavobacteriaceae*. At the adult stage, diet regime (sugar meal and blood meal) significantly affects the microbial structure. Intriguingly, blood meals drastically reduced the community diversity and favored enteric bacteria. Comparative genomic analysis revealed that the enriched enteric bacteria possess large genetic redox capacity of coping with oxidative and nitrosative stresses that are associated with the catabolism of blood meal, suggesting a beneficial role in maintaining gut redox homeostasis. Interestingly, gut community structure was similar in the adult stage between the field and laboratory mosquitoes, indicating that mosquito gut is a selective eco-environment for its microbiome. This comprehensive gut metatgenomic profile suggests a concerted symbiotic genetic association between gut inhabitants and host.

## Introduction

Rapid advances in metagenomics have revived interest in the symbiotic interactions between hosts and their associated gut microbial assemblages [1], [2], [3], and results of these new research avenues are deepening our understanding of how gut microbial communities affect host traits such as metabolism, fecundity, immunity, and longevity [4]. The gut microbiome of mosquitoes can be very dynamic, because mosquitoes shift from aquatic to terrestrial habitats during metamorphosis from larvae through pupae to adults. Additionally, female mosquitoes are hematophagous, a special dietary regime required for egg production. Characterization of microbial community structure is a prerequisite for understanding how a microbial population functions in the gut ecosystem. Several reports using culture dependent and cloning based approaches have shown the diversity of gut microbial fauna in different mosquitoes [5], [6], [7], [8], [9], [10], [11], [12], [13]. However, a comprehensive characterization of gut microbial dynamics across life history is not yet available. In this study, we systematically profiled the gut bacterial communities of different life stages of *Anopheles gambiae*, the primary malaria vector mosquito in Africa, using high throughput pyrosequencing of bacterial 16S rRNA gene fragments. Our data, with unprecedented resolution, revealed previously unperceived community shifts during life stage transitions and community structure associated with dietary regimes, which depicted the metagenomic landscape in the mosquito gut ecosystem for the first time.

## Results and Discussion

### Dynamic structure of gut microbial communities across life stages

**Figure 1:**
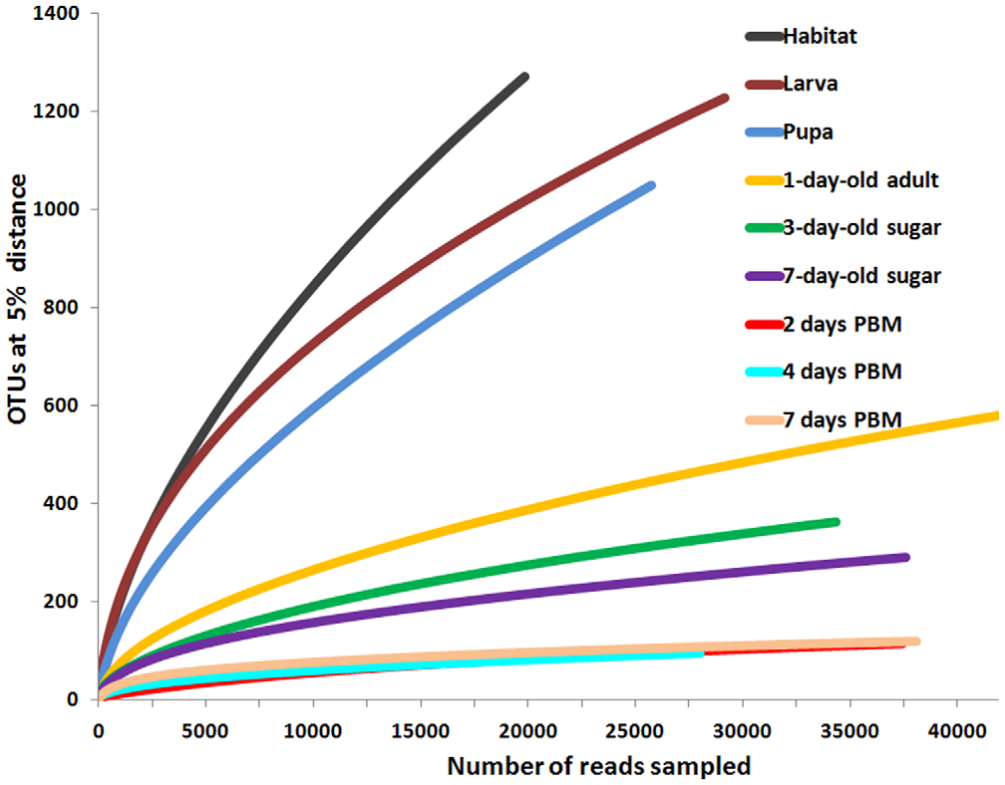
Rarefaction analysis for each sample. OTU s at 5% distance for each site was used to calculate rarefaction curves. Sugar, mosquitoes were fed with sugar meal; PBM, post blood meal.

**Figure 2:**
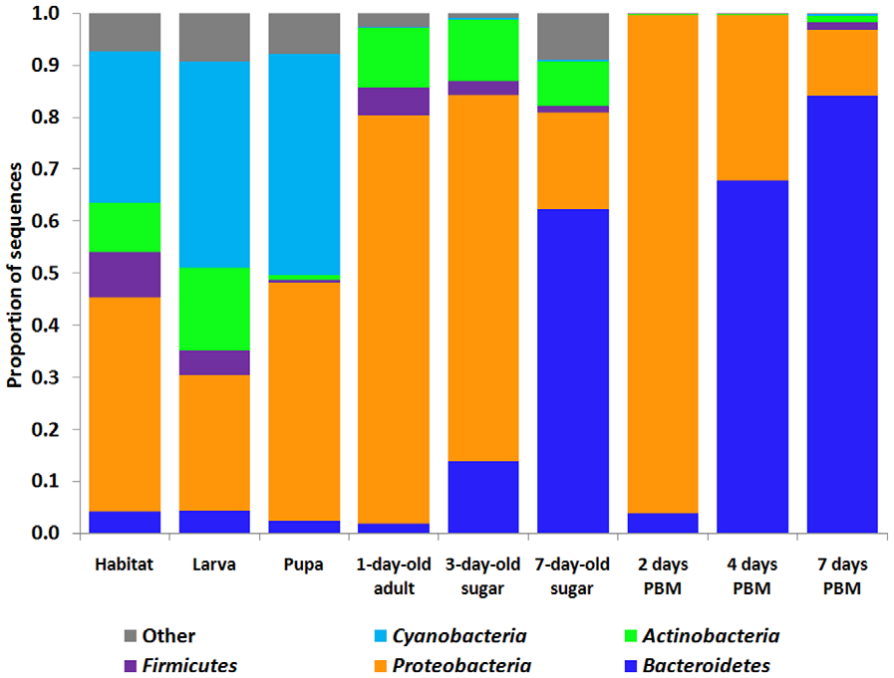
Gut bacterial composition at phylum level in different life stages of *An. gambiae* in Kenya. Phyla *Cyanobacteria*, *Proteobacteria*, *Bacteroidetes*, *Actinobacteria* and *Firmicutes* are presented. Other phyla were pooled as “Other”. PBM, post blood meal. Refer Figure S1 for the details of sample collection.

**Figure 3:**
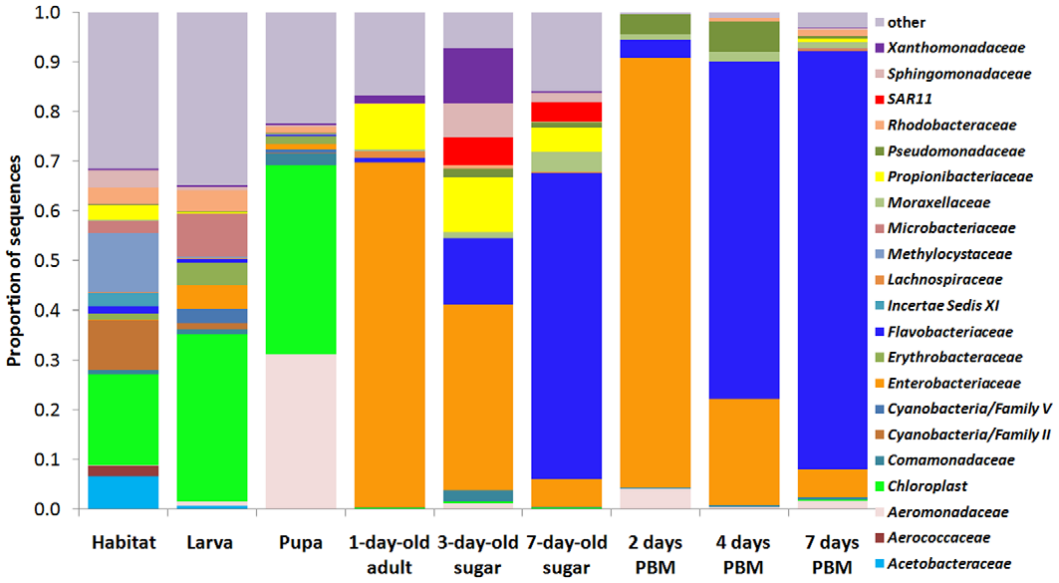
Gut bacterial composition at family level in different life stages of *An. gambiae* in Kenya. Families with abundance of ≥2% in at least one sample are presented. Taxa with abundance of <2% were pooled together as “Other”. Sugar, fed with sugar meal; PBM, post blood meal.

Mosquitoes were reared in semi-natural habitats made with local topsoil and rain water in Kisian, Kenya. Three experiments were conducted. In each experiment, nine samples were collected, including larval habitat surface water, fourth instar larvae, pupae, and six adult gut samples from sugar fed and blood fed mosquito at different time points (Figure S1). To survey microbial communities, bacterial 16S rRNA gene V1–V3 variable regions (positions 27–519) were PCR amplified and pyrosequenced for each sample. Approximately 650,000 sequence reads were obtained from all 27 collections. 426,324 sequences were used for further analysis after removing sequences shorter than 300 base pairs and potential chimera sequences. Species richness and diversity estimators ACE and Chao1 were calculated based on Operational Taxonomic Units (OTUs) implemented in the software Mothur [14], and are summarized in Table S1. The dynamic gut community complexity was demonstrated in rarefaction curves (Figure 1). Habitat water samples had the highest species diversity with the largest number of OTUs while in larvae and pupae fewer OTUs were detected, suggesting that only a subset of bacteria from the aquatic habitat were able to inhabit the mosquito gut. The number of OTUs further decreased in the guts of adults fed on a sugar meal. The lowest diversity was observed in the guts of blood-fed mosquitoes (Table S1). The rarefaction curves leveled off in the adult gut samples, indicating that adequate sampling had been achieved and most OTUs had been retrieved (Figure 1). However, more OTUs might be detected if more sequences were sampled in the larval and pupal collections. We then characterized the taxonomic composition and relative abundance of taxa across samples. A classifier at the Ribosomal Database Project (RDP) server [15] was used to assign reads to appropriate taxonomic ranks. A total of 18 phyla, 138 families and 337 genera were identified. About 80% of sequence reads were able to be assigned to general level, and the remaining 20% were unassignable to a certain rank, suggesting the existence of previously uncharacterized taxa. The phyla *Cyanobacteria*, *Proteobacteria*, *Bacteroidetes*, *Actinobacteria* and *Firmicutes* together constituted 90.7–99.9% of the bacterial communities across all life stages (Figure 2). Detailed community composition and relative abundance at levels of phylum, family and genus are presented in Figure 3 and Table S2, S3, and S4. In the larval and pupal stages, photosynthetic *Cyanobacteria* were prominently abundant, representing ∼40% of the communities, consistent with previous reports [16], [17]. During the transition from pupa to adult, gut community structure changed drastically. For instance, *Chloroplast* in *Cyanobacteria* and *Aeromonadaceae*, *Comamonadaceae*, *Erythrobacteraceae* and *Rhodobacteraceae* in *Proteobacteria* that represented 74.4% of the community in pupae dropped to low or undetectable levels in newly emerged adults (24 hr post-eclosion, no sugar feeding). The places were taken by families *Enterobacteriaceae* and *Propionibacteriaceae*. The differential abundance of these taxa between the two samples was statistically significant (Table S5). Remarkably *Enterobacteriaceae* accounted for 69.4% of the community at this time point, dominated by *Thorsellia anophelis* [18], a species originally isolated from the gut of the mosquito *An. arabiensis* [9] and found in wild caught *An. gambiae* [19]. It has been demonstrated that during metamorphosis pupal midgut contents including sloughed larval midgut epithelial cells and microbes are encased in meconial peritrophic matrices, which will be egested around 24 hr post emergence [20], [21]. This process is believed to sequestrate and scavenge gut microbes. In addition, bacteria can be transstadially transferred from pupae to adults [22]. The prominent switch of bacterial community structure from pupae to adults may be attributed to the processes as well as different gut environment conditions between larvae and adults [23]. In the two collections of sugar fed guts at day 3 and day 7 post eclosion, *Flavobacteriaceae* in *Bacteroidetes* progressively increased from 13.2 to 61.7% while *Enterobacteriaceae* decreased from 37.4 to 5.5%. The abundance of family *SAR11* in α-*Proteobacteria* was relatively stable (5.6% and 3.8%, respectively). A blood meal substantially reduced the community diversity, and *Proteobacteria* occupied nearly the whole community (Figure 2). In the 3-day-old sugar-fed gut, 10 families represented 92.0% of the community. In contrast, in the gut at 2 days post blood meal 4 families (*Enterobacteriaceae* (86.6%), *Aeromonadaceae* (4.2%) and *Pseudomonadaceae* (4.0%)) accounted for 94.8% of the community (Figure 3, Table S3). The difference in abundance of these taxa before and after blood feeding was statistically significant (Table S6). Noticeably, *Elizabethkingia* in *Flavobacteriaceae* became dominant in the guts of 7-day-old (sugar fed) and 4 and 7 days post blood feeding (Table S4). *Elizabethkingia meningoseptica* had been repeatedly detected in both lab and wild caught mosquitoes [10], [22], [24], indicating its prevalent symbiotic association with mosquitoes.

### Nutrient provisions, redox status and community shifts

It is likely that gut conditions affect bacterial community structure. Nutritional provisions in the gut differ between sugar fed and blood fed mosquitoes. Due to limited energy reserves, newly emerged females need to feed to replenish energy in order to power flight for swarming, mating and host-seeking activities [25], [26]. Nectar has been shown to be a preferred choice for the first meal prior to blood meals [27], [28], [29], [30]. Nectar is largely composed of carbohydrates and a few free amino acids [31], which satisfy the flight energy demand as mosquitoes can use carbohydrates [32] and the free amino acid proline [33] to fuel flight. Nutritionally, a sugar fed gut is carbohydrate-rich but protein limited. A blood meal transforms the gut into a protein-rich environment and alters the metabolic architecture in the gut. Such changes are concurrent with compositional shifts in the microbiome. For example, bacteria in the family *SAR11* were persistently housed in the sugar-fed gut, but were undetectable after blood feeding (Figure 3, Table S3, S6). The *SAR11* family was first found in oligotrophic ocean water as a ubiquitous, abundant clade in the α-proteobacterial lineage [34]. *Pelagibacter ubique*, a representative organism of the *SAR11*, possesses a small streamlined genome of 1.3 Mbp, encoding only minimum metabolic and regulatory functions in adaptation to their nutrient scarce environment [35]. The depletion of *SAR11* bacteria after a blood meal suggests that these organisms are oligotrophs that are able to live in low nutritional environments but lack the capacity to cope with toxic metabolites in nutrient rich environments [36] (see below).

**Figure 4:**
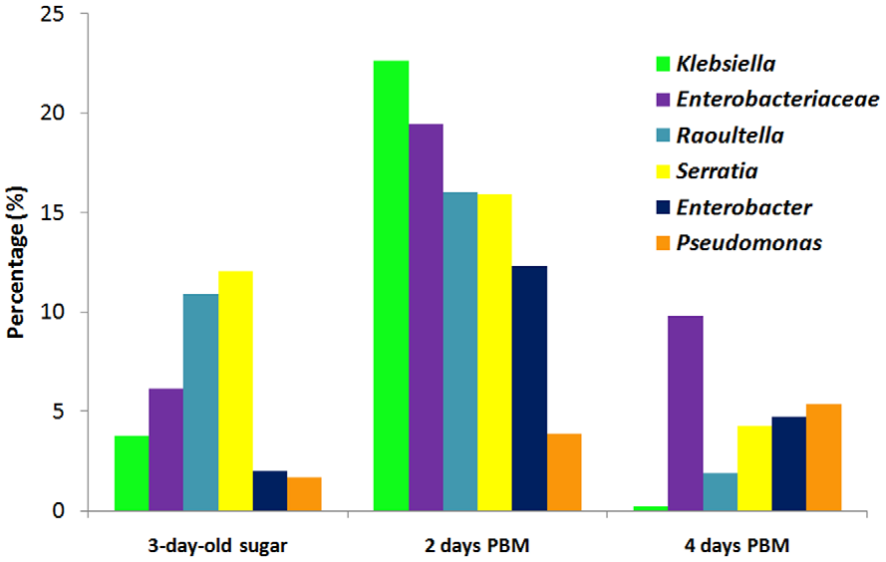
Abundant genera in the gut community after blood feeding. Unclassified *Enterobacteriaceae* represents the reads that were assigned to the family *Enterobacteraceae*, but were not able to be assigned to a genus.

The catabolism of a blood meal results in a large amount of free heme due to the hydrolysis of hemoglobin, leading to the generation of reactive oxygen species (ROS), such as O_2_^−^ and H_2_O_2_ [37], [38], [39]. Blood meals also induce expression of nitric oxide synthase [40], which mediates the production of nitric oxide (NO), a free radical functioning as a source of reactive nitrogen oxide species (RNOS) [40], [41], [42]. Moreover NO reacts with O_2_^−^ to form peroxynitrite. High levels of these toxic oxidants may damage cellular macromolecules, such as DNA, proteins and lipids. Hematophagous mosquitoes have evolved various mechanical and detoxification mechanisms to protect themselves against these toxic oxidants [42], [43]. In response to a blood meal, a peritrophic matrix (PM) is transiently formed to spatially separate the blood bolus from the gut epithelia [44]. The PM has a large heme-binding capacity and traps the released heme [45], [46]. However, bacteria are confined in the endoperitrophic space [11] and exposed directly to the stress associated with blood digestion. Intriguingly, total bacterial load increased quickly after a blood meal (Figure S2) [6], [11], [47], and the community was reshaped with reduction in diversity and expansion of *Enterobacteriaceae* (Figure 3). In the family, genera *Serratia* (15.9%), *Raoultella* (16.0%), *Enterobacter* (12.3%), *Klebsiella* (22.6%) and unidentifiable taxa (19.4%) together accounted for 86.2% of the community. In addition, *Pseudomonas* also increased to 3.9% (Figure 4, Table S4). The differential abundance of these taxa before and after blood feeding was significant statistically (Table S7). These dominant bacteria are likely capable of coping with the oxidative stresses in the bolus. Presumably the restructured community following blood feeding may be related to bacterial anti-stress capability. To examine this hypothesis, we used comparative genomics to analyze the stress response system in representative bacterial genomes. We chose bacterial species that are closely related to the gut habitants as shown by 16S rRNA gene similarity (Figure S3). These representative bacteria are *Serratia marcescens*, *Klebsiella pneumonia*, *Enterobacter cloacae*, *Pseudomonas aeruginosa*, *Pelagibacter ubique* and *Propionibacterium acnes*. The features of the stress response subsystem present in the six genomes are shown in Table S8. Bacterial antioxidant responses are primarily coordinated by two regulons, SoxR and OxyR [48]. In enterobacteria, SoxR senses O_2_^−^ and activates SoxS, a general transcription regulator that controls a variety of genes involved in antioxidant production and oxidative damage repair as well as genes involved in multidrug resistance and heavy metal detoxification [49], [50], including superoxide dismutase. OxyR is a sensor of H_2_O_2_ and controls the activation of major peroxide-degrading enzymes, including catalase, peroxiredoxin, thioredoxin reductase, and other genes responding to oxidative stress [51], [52], [53], [54]. On the other hand, nitrosative stress is sensed by NO sensors NorR and NsrR and FNR [55]. NorR and Fur transcribe genes encoding flavorubredoxin and associated flavoprotein, which together reduce NO to N_2_O under aerobic conditions [56], [57], [58]. Flavohemoglobins (flavoHbs) are made of a globin domain fused with a ferredoxin reductase-like FAD- and NAD-binding modules, which play a crucial role in the NO homeostasis [59], [60], [61]. The expression of flavoHbs is regulated by FNR and NsrR responding to NO challenge [62], [63]. Aerobically, flavoHbs detoxify NO as a NO dioxygenase by reducing NO to NO_3_^−^ [64], [65], and anaerobically flavoHbs may act as NO reductase to convert NO to N_2_O [66]. These genes constitute a large reducing capacity to tackle oxidative and nitrosative stresses. As shown in Table S8, of 170 genes assigned to the stress response subsystem 81–117 genes are present in the three representative enterobacteria and *P. aeruginosa*. These and their relatives were expanded in blood fed gut (Figure 4). In contrast, *P.acnes* and *P.ubique*, the relatives of which were rare or undetectable after feeding, have only 10 and 27 genes, respectively. Interestingly, both *P. acnes* and *P. ubique* have catalase and manganese superoxide dismutase, but lack genes protecting from nitrosative stress, implying that NO-associated stress might be more important in shaping the microbiome structure.

The blood fed gut has been shown to be a reducing environment [41]. The presence of an expanded bacterial redox reservoir during blood digestion could be one of the essential factors maintaining gut redox homeostasis. In fact, in the blood fed gut, bacterial proliferation is enabled by a dityrosine network that is formed by a dual oxidase/peroxidase involved mechanism. The crosslinked mucus layer at the lumenal side of the epithelium reduces the permeability to bacterial immune elicitors, which allows bacterial growth in the endoperitrophic space without activating host anti-microbial response [67]. Consistently, recent evidence shows that in blood fed *Aedes* mosquitoes, released heme decreases ROS production in the midgut cells, which occurs concomitantly with the expansion of gut bacterial load [68]. The transient bacterial expansion subsided when blood digestion was over; the bacterial abundance at 4 days post feeding was similar to the level prior to feeding (Figure S2).

It has been shown that mosquito gut commensal bacteria prime basal immunity. Perturbation of the microbial community compromises mosquito immunity against malaria [69]. Further, our data suggests that gut bacterial redox capacity could keep the milieu in a reducing state, which is critical for the anti-malarial nitrogen oxides to be produced [40], [41].

Besides the possibility discussed above, other factors may also contribute to the shift of community structure. For example, expanded enteric bacteria may be resistant to the digestion enzymes, and/or they possess mechanisms to avoid to be egested. Further study with metatranscriptomic analysis would provide information to assess these possibilities.

### Community comparison between Kenyan and laboratory mosquitoes

To assess the similarity of gut bacterial consortia between *An. gambiae* from Kenya and laboratory, we compared the community structures across aquatic stages, sugar fed and blood fed adults. Bacterial community structure was less similar in the aquatic stages (habitat, larva and pupa), but became more comparable in the adult stages. At family level, 57.6–88.0% of taxa that were detected in lab mosquitoes were also present in Kenyan samples, and these shared taxa represented 57.8–99.7% of the total reads in the lab mosquitoes. In the Kenyan communities 18.9–45.2% of the families were shared with the lab counterparts, representing 20.9%–95.2% of total reads in the communities (Table 1, S9). In other words, most taxa found in the lab conditions existed in the Kenyan samples, and these shared taxa represented the majority of the communities. For example, at 2 days post blood feeding, *Enterobacteriaceae*, *Flavobacteriaceae* and *Pseudomonadaceae* together represented 80.9% and 94.3% of the community in lab and Kenyan samples, respectively. The comparable community structure in adults, especially after blood meals, between laboratory and Kenyan mosquitoes is intriguing, strongly suggesting that the core bacterial symbionts, mostly from *Enterobacteriaceae* and *Flabobacteriaceae*, are naturally associated with the mosquito gut environment and independent of external conditions. From the perspective of paratransgenesis, steady associated abundant bacteria have the potential to be used to develop symbiotic carriers to deliver desired molecules for the control of mosquito-borne diseases [70], [71].

**Table 1:** Comparison of community structure between laboratory and Kenyan samples.

Samples	Shared No./total No. of families (%)		% of reads in shared families vs total reads in the community	
	Lab	Kenya	Lab	Kenya
Habitat	25/30(83.33)	25/62(40.32)	57.84	20.93
Larva	44/50 (88.00)	44/119 (36.97)	97.39	52.85
Pupa	30/38 (78.95)	30/80 (37.50)	95.33	44.57
1-day-old adult, no feeding	32/42 (76.19)	32/75 (42.67)	95.30	86.58
3-day-old, sugar fed	19/33 (57.58)	19/42 (45.24)	95.22	79.71
7-day-old, sugar fed	10/14 (71.43)	10/53 (18.87)	93.65	73.76
2 days post blood meal	8/14 (57.14)	8/36 (22.22)	83.08	95.16
4 days post blood meal	8/11 (72.73)	8/42 (19.05)	99.74	95.04

Refer Table S9 for composition details.

The current study revealed a dynamic mosquito gut microbiome that undergoes significant shifts in composition concomitantly with transitions of host life history. Our findings emphasize the need to link gut ecological structure to its genetic functionality that affects host traits. Blood feeding is crucial for mosquito fecundity and transmission of mosquito-borne diseases. The intriguing impact of blood meals on reshaping the gut community with the bacteria having large antioxidant capacity suggests a beneficial symbiosis in the gut ecosystem. From an ecological point of view, proper functionality of a microbiome is a matter of compositional harmony of the consortium. Intentionally disturbing microbial community structure to achieve dysbiosis of the mosquito gut ecosystem is a new avenue to be explored for developing measures for mosquito control.

## Materials and Methods

### Ethics Statement

The animal usage followed the protocol (number 2008-031) that was approved by the New Mexico State University Institutional Animal Care and Use Committee.

### Microcosm setup and study site

The semi-natural microcosms were established at the Kenya Medical Research Institute (KEMRI) in Kisian, Kenya, in May and September, 2010. The microcosms were constructed using 60 cm diameter washtubs to which approximately four liters of local top-soil were added. Rainwater was collected from roof runoff and approximately six liters were added to each microcosm. Microcosms were placed in full sun, screened with a light mesh to prevent oviposition by wild mosquitoes and left for five days to allow for microbial colonization prior to addition of larvae. A colony of *Anopheles gambiae* s.s. maintained in the insectary in Kisian was used as the source of larvae. Five hundred first instar larvae were added to each microcosm. Water levels were maintained as needed by the addition of rainwater. Microcosms were monitored daily and any pupae present were removed for emergence in the insectary.

### Sample collection

Thirty fourth instar larvae were removed prior to pupation, washed three times with sterile water, rinsed twice with 100% ethanol and stored in 100% ethanol until further processing. On the same day, 50 ml surface microlayer (∼2 mm) water samples were collected with needles and 50 ml syringes. Water samples were centrifuged three times at 12,800× g and the resultant pellet was stored in 100% ethanol until processing. Thirty pupae were also taken from microcosms and stored in 100% ethanol until processing. Remaining pupae were divided equally and transferred to cages daily. Thirty newly emerged, unfed adults were removed from the cages at approximately 24 hrs and adult guts were removed and stored in 100% ethanol. In all dissections, dissecting tools were sterilized. Before dissection, adult specimens were surface-cleaned by rinsing three times, 5 seconds each time, sequentially in three 5-ml tubes containing 100% ethanol. Adult guts were removed and rinsed three times in 100% ethanol and then stored in 100% ethanol until further processing. One half of the remaining adults were supplied with sugar for the remainder of the experiment, and one half were fed on rabbit for a blood meal at 72 hrs post emergence. Thirty adult guts were removed, rinsed and stored in 100% ethanol at 3 and 7 days post-emergence (sugar-fed) and 2, 4 and 7 days post blood feeding. The collection scheme is summarized in Figure S1. All water, larval, pupal, and adult gut samples were taken in triplicate from three experiments. Samples were sent to Research and Testing Laboratory (Lubbock, TX) for further processing.

### Laboratory mosquitoes

*Anopheles gambiae* G3 strain was obtained from MR4, and was maintained in an insectary at 27°C with 12/12 hr day-night light cycle. Larval food consisted of Brewer's yeast, lactalbumin and rat chow powder (1∶1∶1). Adults were provided with 10% sucrose solution, and for the egg production, adults were fed on NIH Swiss outbred mice for a blood meal at 3 days post-emergence. To compare the gut microbiome with the Kenyan counterparts, corresponding samples in triplicate at each point were collected using the same procedure described above.

### Bacterial 16S rDNA PCR, pyrosequencing, sequence analysis and statistics

The bacterial composition was surveyed by using sequences of taxon proxy 16S rRNA gene fragments [72]. Variable region V1–3 was amplified with primers 27F 5′ GAG TTT GAT CNT GGC TCA G 3′ and 519R 5′ GTN TTA CNG CGG CKG CTG 3′. The template DNA preparation, 16S rRNA gene PCR and pyrosequencing were conducted at Research and Testing Laboratories using the Roche Titanium sequencing platform as described previously [73], [74]. A total of 668,286 reads with 200–550 bp in length were generated from Kenyan samples. Sequences were deposited in the NCBI sequence reads archive (accession number SRA031282.2). To estimate species richness and diversity across communities, the operational taxonomic unit (OTU) based analysis was used and implemented using software Mothur [14], [75]. The sequence set was depleted of short reads (<300 bp) and potential chimera sequences were excluded using ChimeraSlayer (http://microbiomeutil.sourceforge.net/) implemented in the Mothur (v.1.16). The resultant 426,324 sequences were used for further analysis. Due to the limitation of computational capacity (4 CPUs and 16G RAM), we pooled reads from triplicates of each collection point and randomly selected up to 50,000 reads for community diversity estimation using Mothur. The sequence reads were aligned against SILVA [76] reference alignment and clustered into groups of defined sequence variation that ranged from 3% to 10% differences. These clusters served as OTUs for generating rarefaction curves and for making calculations with the abundance-based coverage estimator ACE [77], [78] and the species diversity estimator Chao1 [79]. Rarefaction analysis uses resampling of community survey data to generate idealized collector's curves, an indication of overall species richness [80]. Therefore, rarefaction can provide an estimate of the depth to which a complex community has been sampled. To estimate community composition the taxon based analysis was used. The reads were assigned to taxonomic rank using a web-based RDP classifier [15] with confidence threshold 80%. Using V3 region, the cutoff warrants a reliability of 98.1% to correctly classify a read to genus (http://rdp.cme.msu.edu/classifier/class_help.jsp#format). Taxon composition and relative abundance were calculated separately for three samples of each collection point. The average percentage of taxa was presented. The differential abundant taxa between collections were detected with Metastats at their web server (http://metastats.cbcb.umd.edu/detection.html), a statistical method assessing taxonomic difference in metagenomic samples [81].

### Quantitation of gut bacterial load

A Real-Time quantitative PCR method detecting the amount of 16S rRNA copies was developed for measuring bacterial load. 16S rRNA gene fragment was amplified with primers 27F 5′GAG TTT GAT CNT GGC TCA G 3′ and 342R 5′CTG CTG CCT CCC GTA G 3′. Mosquito gene rpS7 was used as a reference gene for the DNA loading control, which was amplified using primers forward 5′CCA TCC TGG AGG ATC TGG TA 3′ and reverse 5′ GAT GGT GGT CTG CTG GTT CT 3′. Real-time PCR was run using genomic DNA template at the Roche LightCycler 480 (Roche Applied Sciences, Indianapolis, IN) following the instruction of manufacturer. Bacterial load is presented as ratio of 16S vs rpS7.

### Bacterial genomic comparison

Six representative bacterial genomes were compared for their capacity in the oxidative stress subsystem. The genomic sequences of *Enterobacter cloacae* subsp. cloacae ATCC 13047 (Project ID 45793), *Klebsiella pneumoniae* 342 (ID 28471) *Pseudomonas aeruginosa* 2192 (ID 16171), *Propionibacterium acnes* KPA171202 (ID 12460), *Candidatus Pelagibacter ubique* (ID 15602) were obtained from the NCBI genome database. The genome sequences of *Serratia marcescens* Db11 was obtained from the Sanger Institute. The oxidative stress subsystem of these genomes was annotated at the RAST (Rapid Annotation using Subsystem Technology) server, and displayed on the SEED Viewer [82]. The sequence relatedness of the abundant taxa in the gut collections to the above representative species is presented in Fig. S2. The reads that were assigned to the *Enterobacter*, *Serratia*, *Klebsiella*, *Pseudomonas*, *Propionibacterium* and *SAR11/Pelagibacter* were aligned with the SeqMan program in the DNAStar software (DNAStar, Inc., Madison, WI). The consensus sequences of the contigs assembled from abundant reads were used for relatedness analysis. The 16S rDNA sequences of representative species were trimmed, and the region spanning V1–V3 were used for the analysis. The resultant sequences were analyzed using software MEGA 5.02 [83]. The phylogenetic relatedness was reconstructed with Maximum Likelihood (ML) and Neighbor Joining (NJ) methods, and both yielded trees with similar topology. The NJ tree in Figure S3 was constructed with Kimura-2 parameter model, gamma distribution of rate among sites, bootstrap with 1000 replications.

## Supporting Information

Figure S1 **Sample collection scheme for field experiments in Kenya.** Each box represents asample collection point. PBM: post blood meal.
(TIF)

Figure S2 **Bacterial abundance in mosquito guts before and after a blood meal.** The relative abundance of 16S rDNA is presented as Mean±SD from three replicates. Sugar, sugar fed; PBM, post blood meal.
(TIF)

Figure S3 **Phylogenetic relatedness of representative bacterial species and mosquito gut bacteria based on 16S rRNA tag sequence similarity.** Neighbor-joining tree is presented. Bootstrap values (>50) are given at nodes. Representative bacteria are presented in bold fonts.
(TIF)

Table S1 **Similarity-based OTUs and species richness estimates.**
(PDF)

Table S2 **Gut bacterial composition at phylum level across life stages of** ***An.gambiae*** **.**
(PDF)

Table S3 **Gut bacterial composition at family level across life stages of** ***An.gambiae*** **.**
(PDF)

Table S4 **Gut bacterial composition at genus level across life stages of** ***An.gambiae*** **.**
(PDF)

Table S5 **Differentially abundant families between pupal and newly emerged adult guts.**
(PDF)

Table S6 **Differentially abundant families before and after a blood meal.**
(PDF)

Table S7 **Differentially abundant genera before and after a blood meal.**
(PDF)

Table S8 **Genomic comparison on anti-stress subsystems in abundant bacteria species from blood fed and sugar fed guts.**
(PDF)

Table S9 **Comparison of gut bacterial composition at family level between Kenyan and lab samples.**
(PDF)

## References

[1] Warnecke F, Luginbuhl P, Ivanova N, Ghassemian M, Richardson TH (2007). Metagenomic and functional analysis of hindgut microbiota of a wood-feeding higher termite.. Nature.

[2] Tartar A, Wheeler MM, Zhou X, Coy MR, Boucias DG (2009). Parallel metatranscriptome analyses of host and symbiont gene expression in the gut of the termite Reticulitermes flavipes.. Biotechnol Biofuels.

[3] Suen G, Scott JJ, Aylward FO, Adams SM, Tringe SG (2010). An insect herbivore microbiome with high plant biomass-degrading capacity.. PLoS Genet.

[4] Dillon RJ, Dillon VM (2004). The gut bacteria of insects: nonpathogenic interactions.. Annu Rev Entomol.

[5] Gonzalez-Ceron L, Santillan F, Rodriguez MH, Mendez D, Hernandez-Avila JE (2003). Bacteria in midguts of field-collected Anopheles albimanus block Plasmodium vivax sporogonic development.. J Med Entomol.

[6] Pumpuni CB, Demaio J, Kent M, Davis JR, Beier JC (1996). Bacterial population dynamics in three anopheline species: the impact on Plasmodium sporogonic development.. Am J Trop Med Hyg.

[7] Straif SC, Mbogo CN, Toure AM, Walker ED, Kaufman M (1998). Midgut bacteria in Anopheles gambiae and An. funestus (Diptera: Culicidae) from Kenya and Mali.. J Med Entomol.

[8] Terenius O, de Oliveira CD, Pinheiro WD, Tadei WP, James AA (2008). 16S rRNA gene sequences from bacteria associated with adult Anopheles darlingi (Diptera: Culicidae) mosquitoes.. J Med Entomol.

[9] Lindh JM, Terenius O, Faye I (2005). 16S rRNA gene-based identification of midgut bacteria from field-caught Anopheles gambiae sensu lato and A. funestus mosquitoes reveals new species related to known insect symbionts.. Appl Environ Microbiol.

[10] Rani A, Sharma A, Rajagopal R, Adak T, Bhatnagar RK (2009). Bacterial diversity analysis of larvae and adult midgut microflora using culture-dependent and culture-independent methods in lab-reared and field-collected Anopheles stephensi-an Asian malarial vector.. Bmc Microbiology.

[11] Gusmao DS, Santos AV, Marini DC, Bacci M, Berbert-Molina MA (2010). Culture-dependent and culture-independent characterization of microorganisms associated with Aedes aegypti (Diptera: Culicidae) (L.) and dynamics of bacterial colonization in the midgut.. Acta Trop.

[12] Pidiyar VJ, Jangid K, Patole MS, Shouche YS (2004). Studies on cultured and uncultured microbiota of wild culex quinquefasciatus mosquito midgut based on 16s ribosomal RNA gene analysis.. Am J Trop Med Hyg.

[13] Damiani C, Ricci I, Crotti E, Rossi P, Rizzi A (2010). Mosquito-bacteria symbiosis: the case of Anopheles gambiae and Asaia.. Microb Ecol.

[14] Schloss PD, Westcott SL, Ryabin T, Hall JR, Hartmann M (2009). Introducing mothur: open-source, platform-independent, community-supported software for describing and comparing microbial communities.. Appl Environ Microbiol.

[15] Wang Q, Garrity GM, Tiedje JM, Cole JR (2007). Naive Bayesian classifier for rapid assignment of rRNA sequences into the new bacterial taxonomy.. Appl Environ Microbiol.

[16] Vazquez-Martinez MG, Rodriguez MH, Arredondo-Jimenez JI, Mendez-Sanchez JD, Bond-Compean JG (2002). Cyanobacteria associated with Anopheles albimanus (Diptera: Culicidae) larval habitats in southern Mexico.. J Med Entomol.

[17] Thiery I, Nicolas L, Rippka R, Tandeau de Marsac N (1991). Selection of cyanobacteria isolated from mosquito breeding sites as a potential food source for mosquito larvae.. Appl Environ Microbiol.

[18] Kampfer P, Lindh JM, Terenius O, Haghdoost S, Falsen E (2006). Thorsellia anophelis gen. nov., sp. nov., a new member of the Gammaproteobacteria.. Int J Syst Evol Microbiol.

[19] Briones AM, Shililu J, Githure J, Novak R, Raskin L (2008). Thorsellia anophelis is the dominant bacterium in a Kenyan population of adult Anopheles gambiae mosquitoes.. ISME J.

[20] Moll RM, Romoser WS, Modrzakowski MC, Moncayo AC, Lerdthusnee K (2001). Meconial peritrophic membranes and the fate of midgut bacteria during mosquito (Diptera: Culicidae) metamorphosis.. J Med Entomol.

[21] Romoser WS, Moll RM, Moncayo AC, Lerdthusnee K (2000). The occurrence and fate of the meconium and meconial peritrophic membranes in pupal and adult mosquitoes (Diptera: Culicidae).. J Med Entomol.

[22] Lindh JM, Borg-Karlson AK, Faye I (2008). Transstadial and horizontal transfer of bacteria within a colony of Anopheles gambiae (Diptera: Culicidae) and oviposition response to bacteria-containing water.. Acta Trop.

[23] Linser PJ, Smith KE, Seron TJ, Neira Oviedo M (2009). Carbonic anhydrases and anion transport in mosquito midgut pH regulation.. J Exp Biol.

[24] Kajla MK, Andreeva O, Gilbreath TM, Paskewitz SM (2010). Characterization of expression, activity and role in antibacterial immunity of Anopheles gambiae lysozyme c-1.. Comp Biochem Physiol B Biochem Mol Biol.

[25] Foster WA (1995). Mosquito sugar feeding and reproductive energetics.. Annu Rev Entomol.

[26] Briegel H (1990). Fecundity, metabolism, and body size in Anopheles (Diptera: Culicidae), vectors of malaria.. J Med Entomol.

[27] Foster WA, Takken W (2004). Nectar-related vs. human-related volatiles: behavioural response and choice by female and male Anopheles gambiae (Diptera: Culicidae) between emergence and first feeding.. Bull Entomol Res.

[28] Gary RE, Foster WA (2004). Anopheles gambiae feeding and survival on honeydew and extra-floral nectar of peridomestic plants.. Med Vet Entomol.

[29] Impoinvil DE, Kongere JO, Foster WA, Njiru BN, Killeen GF (2004). Feeding and survival of the malaria vector Anopheles gambiae on plants growing in Kenya.. Med Vet Entomol.

[30] Manda H, Gouagna LC, Nyandat E, Kabiru EW, Jackson RR (2007). Discriminative feeding behaviour of Anopheles gambiae s.s. on endemic plants in western Kenya.. Med Vet Entomol.

[31] Gonzalez-Teuber M, Heil M (2009). Nectar chemistry is tailored for both attraction of mutualists and protection from exploiters.. Plant Signal Behav.

[32] Sacktor B, Wormser-Shavit E (1966). Regulation of Metabolism in Working Muscle in Vivo.. Journal of Biological Chemistry.

[33] Scaraffia PY, Wells MA (2003). Proline can be utilized as an energy substrate during flight of Aedes aegypti females.. J Insect Physiol.

[34] Morris RM, Rappe MS, Connon SA, Vergin KL, Siebold WA (2002). SAR11 clade dominates ocean surface bacterioplankton communities.. Nature.

[35] Giovannoni SJ, Tripp HJ, Givan S, Podar M, Vergin KL (2005). Genome streamlining in a cosmopolitan oceanic bacterium.. Science.

[36] Koch AL (2001). Oligotrophs versus copiotrophs.. Bioessays.

[37] Kumar S, Christophides GK, Cantera R, Charles B, Han YS (2003). The role of reactive oxygen species on Plasmodium melanotic encapsulation in Anopheles gambiae.. Proc Natl Acad Sci U S A.

[38] Souza AV, Petretski JH, Demasi M, Bechara EJ, Oliveira PL (1997). Urate protects a blood-sucking insect against hemin-induced oxidative stress.. Free Radic Biol Med.

[39] Toh SQ, Glanfield A, Gobert GN, Jones MK (2010). Heme and blood-feeding parasites: friends or foes?. Parasit Vectors.

[40] Luckhart S, Vodovotz Y, Cui L, Rosenberg R (1998). The mosquito Anopheles stephensi limits malaria parasite development with inducible synthesis of nitric oxide.. Proc Natl Acad Sci U S A.

[41] Peterson TM, Gow AJ, Luckhart S (2007). Nitric oxide metabolites induced in Anopheles stephensi control malaria parasite infection.. Free Radic Biol Med.

[42] Peterson TM, Luckhart S (2006). A mosquito 2-Cys peroxiredoxin protects against nitrosative and oxidative stresses associated with malaria parasite infection.. Free Radic Biol Med.

[43] Graca-Souza AV, Maya-Monteiro C, Paiva-Silva GO, Braz GR, Paes MC (2006). Adaptations against heme toxicity in blood-feeding arthropods.. Insect Biochem Mol Biol.

[44] Lehane MJ (1997). Peritrophic matrix structure and function.. Annu Rev Entomol.

[45] Devenport M, Alvarenga PH, Shao L, Fujioka H, Bianconi ML (2006). Identification of the Aedes aegypti peritrophic matrix protein AeIMUCI as a heme-binding protein.. Biochemistry.

[46] Pascoa V, Oliveira PL, Dansa-Petretski M, Silva JR, Alvarenga PH (2002). Aedes aegypti peritrophic matrix and its interaction with heme during blood digestion.. Insect Biochem Mol Biol.

[47] Demaio J, Pumpuni CB, Kent M, Beier JC (1996). The midgut bacterial flora of wild Aedes triseriatus, Culex pipiens, and Psorophora columbiae mosquitoes.. Am J Trop Med Hyg.

[48] Lushchak VI (2011). Adaptive response to oxidative stress: Bacteria, fungi, plants and animals.. Comp Biochem Physiol C Toxicol Pharmacol.

[49] Pomposiello PJ, Bennik MH, Demple B (2001). Genome-wide transcriptional profiling of the Escherichia coli responses to superoxide stress and sodium salicylate.. J Bacteriol.

[50] Dietrich LE, Teal TK, Price-Whelan A, Newman DK (2008). Redox-active antibiotics control gene expression and community behavior in divergent bacteria.. Science.

[51] Zheng M, Aslund F, Storz G (1998). Activation of the OxyR transcription factor by reversible disulfide bond formation.. Science.

[52] Hishinuma S, Ohtsu I, Fujimura M, Fukumori F (2008). OxyR is involved in the expression of thioredoxin reductase TrxB in Pseudomonas putida.. FEMS Microbiol Lett.

[53] Hishinuma S, Yuki M, Fujimura M, Fukumori F (2006). OxyR regulated the expression of two major catalases, KatA and KatB, along with peroxiredoxin, AhpC in Pseudomonas putida.. Environ Microbiol.

[54] Kim SO, Merchant K, Nudelman R, Beyer WF, Keng T (2002). OxyR: a molecular code for redox-related signaling.. Cell.

[55] Spiro S (2006). Nitric oxide-sensing mechanisms in Escherichia coli.. Biochem Soc Trans.

[56] D'Autreaux B, Tucker NP, Dixon R, Spiro S (2005). A non-haem iron centre in the transcription factor NorR senses nitric oxide.. Nature.

[57] Bush M, Ghosh T, Tucker N, Zhang X, Dixon R (2011). Transcriptional regulation by the dedicated nitric oxide sensor, NorR: a route towards NO detoxification.. Biochem Soc Trans.

[58] Mukhopadhyay P, Zheng M, Bedzyk LA, LaRossa RA, Storz G (2004). Prominent roles of the NorR and Fur regulators in the Escherichia coli transcriptional response to reactive nitrogen species.. Proc Natl Acad Sci U S A.

[59] Schopfer MP, Wang J, Karlin KD (2010). Bioinspired heme, heme/nonheme diiron, heme/copper, and inorganic NOx chemistry: *NO((g)) oxidation, peroxynitrite-metal chemistry, and *NO((g)) reductive coupling.. Inorg Chem.

[60] Gardner PR (2005). Nitric oxide dioxygenase function and mechanism of flavohemoglobin, hemoglobin, myoglobin and their associated reductases.. J Inorg Biochem.

[61] Bonamore A, Boffi A (2008). Flavohemoglobin: structure and reactivity.. IUBMB Life.

[62] Cruz-Ramos H, Crack J, Wu G, Hughes MN, Scott C (2002). NO sensing by FNR: regulation of the Escherichia coli NO-detoxifying flavohaemoglobin, Hmp.. EMBO J.

[63] Bodenmiller DM, Spiro S (2006). The yjeB (nsrR) gene of Escherichia coli encodes a nitric oxide-sensitive transcriptional regulator.. J Bacteriol.

[64] Gardner PR, Gardner AM, Martin LA, Salzman AL (1998). Nitric oxide dioxygenase: an enzymic function for flavohemoglobin.. Proc Natl Acad Sci U S A.

[65] Gardner PR, Costantino G, Salzman AL (1998). Constitutive and adaptive detoxification of nitric oxide in Escherichia coli. Role of nitric-oxide dioxygenase in the protection of aconitase.. J Biol Chem.

[66] Poole RK (2005). Nitric oxide and nitrosative stress tolerance in bacteria.. Biochem Soc Trans.

[67] Kumar S, Molina-Cruz A, Gupta L, Rodrigues J, Barillas-Mury C (2010). A peroxidase/dual oxidase system modulates midgut epithelial immunity in Anopheles gambiae.. Science.

[68] Oliveira JH, Goncalves RL, Lara FA, Dias FA, Gandara AC (2011). Blood Meal-Derived Heme Decreases ROS Levels in the Midgut of Aedes aegypti and Allows Proliferation of Intestinal Microbiota.. PLoS Pathog.

[69] Dong Y, Manfredini F, Dimopoulos G (2009). Implication of the mosquito midgut microbiota in the defense against malaria parasites.. PLoS Pathog.

[70] Riehle MA, Moreira CK, Lampe D, Lauzon C, Jacobs-Lorena M (2007). Using bacteria to express and display anti-Plasmodium molecules in the mosquito midgut.. Int J Parasitol.

[71] Coutinho-Abreu IV, Zhu KY, Ramalho-Ortigao M (2010). Transgenesis and paratransgenesis to control insect-borne diseases: current status and future challenges.. Parasitol Int.

[72] Sogin ML, Morrison HG, Huber JA, Mark Welch D, Huse SM (2006). Microbial diversity in the deep sea and the underexplored “rare biosphere”.. Proc Natl Acad Sci U S A.

[73] Dowd SE, Callaway TR, Wolcott RD, Sun Y, McKeehan T (2008). Evaluation of the bacterial diversity in the feces of cattle using 16S rDNA bacterial tag-encoded FLX amplicon pyrosequencing (bTEFAP).. BMC Microbiol.

[74] Andreotti R, Perez de Leon AA, Dowd SE, Guerrero FD, Bendele KG (2011). Assessment of bacterial diversity in the cattle tick Rhipicephalus (Boophilus) microplus through tag-encoded pyrosequencing.. BMC Microbiol.

[75] Schloss PD (2010). The effects of alignment quality, distance calculation method, sequence filtering, and region on the analysis of 16S rRNA gene-based studies.. PLoS Comput Biol.

[76] Pruesse E, Quast C, Knittel K, Fuchs BM, Ludwig W (2007). SILVA: a comprehensive online resource for quality checked and aligned ribosomal RNA sequence data compatible with ARB.. Nucleic Acids Res.

[77] Chao A, Yang MCK (1993). Stopping rules and estimation for recapture debugging with unequal failure rates.. Biometrika.

[78] Chao A, Lee S-M (1992). Estimating the number of classes via sample coverage.. J Am Stat Assoc.

[79] Chao A (1984). Nonparametric estimation of the number of classes in a population.. Scand J Statist.

[80] Gotelli NJ, Colwell RK (2001). Quantifying biodiversity: procedures and pitfalls in the measurement and comparison of species richness.. Ecol Lett.

[81] White JR, Nagarajan N, Pop M (2009). Statistical Methods for Detecting Differentially Abundant Features in Clinical Metagenomic Samples.. PLoS Comput Biol.

[82] Aziz RK, Bartels D, Best AA, DeJongh M, Disz T (2008). The RAST Server: rapid annotations using subsystems technology.. BMC Genomics.

[83] Lehman RM, Lundgren JG, Petzke LM (2009). Bacterial communities associated with the digestive tract of the predatory ground beetle, Poecilus chalcites, and their modification by laboratory rearing and antibiotic treatment.. Microb Ecol.

