# Supplementary figures and images for: Dynamic Gut Microbiome across Life History of the Malaria Mosquito Anopheles gambiae in Kenya
Source: PLoS One. 2011 Sep 21;6(9):e24767. doi: 10.1371/journal.pone.0024767 (PMC3177825; doi:10.1371/journal.pone.0024767)

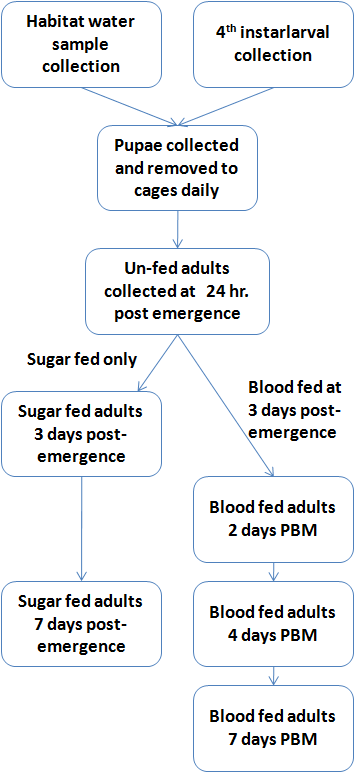

Supplement: Figure S1 — Sample collection scheme for field experiments in Kenya. Each box represents asample collection point. PBM: post blood meal. (TIF) [file pone.0024767.s001.tif]

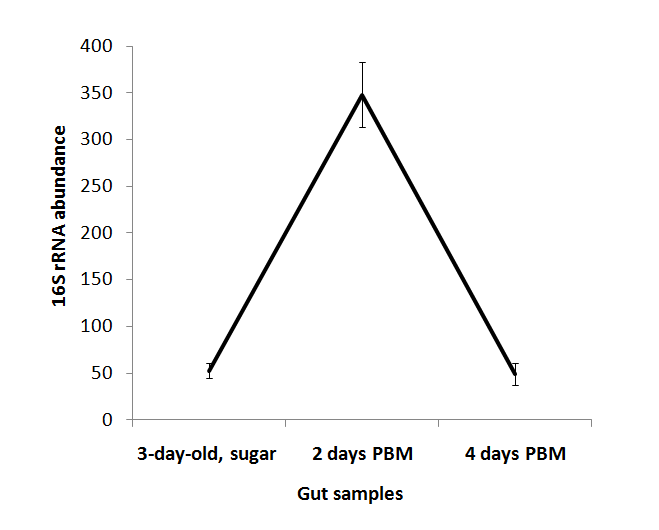

Supplement: Figure S2 — Bacterial abundance in mosquito guts before and after a blood meal. The relative abundance of 16S rDNA is presented as Mean±SD from three replicates. Sugar, sugar fed; PBM, post blood meal. (TIF) [file pone.0024767.s002.tif]

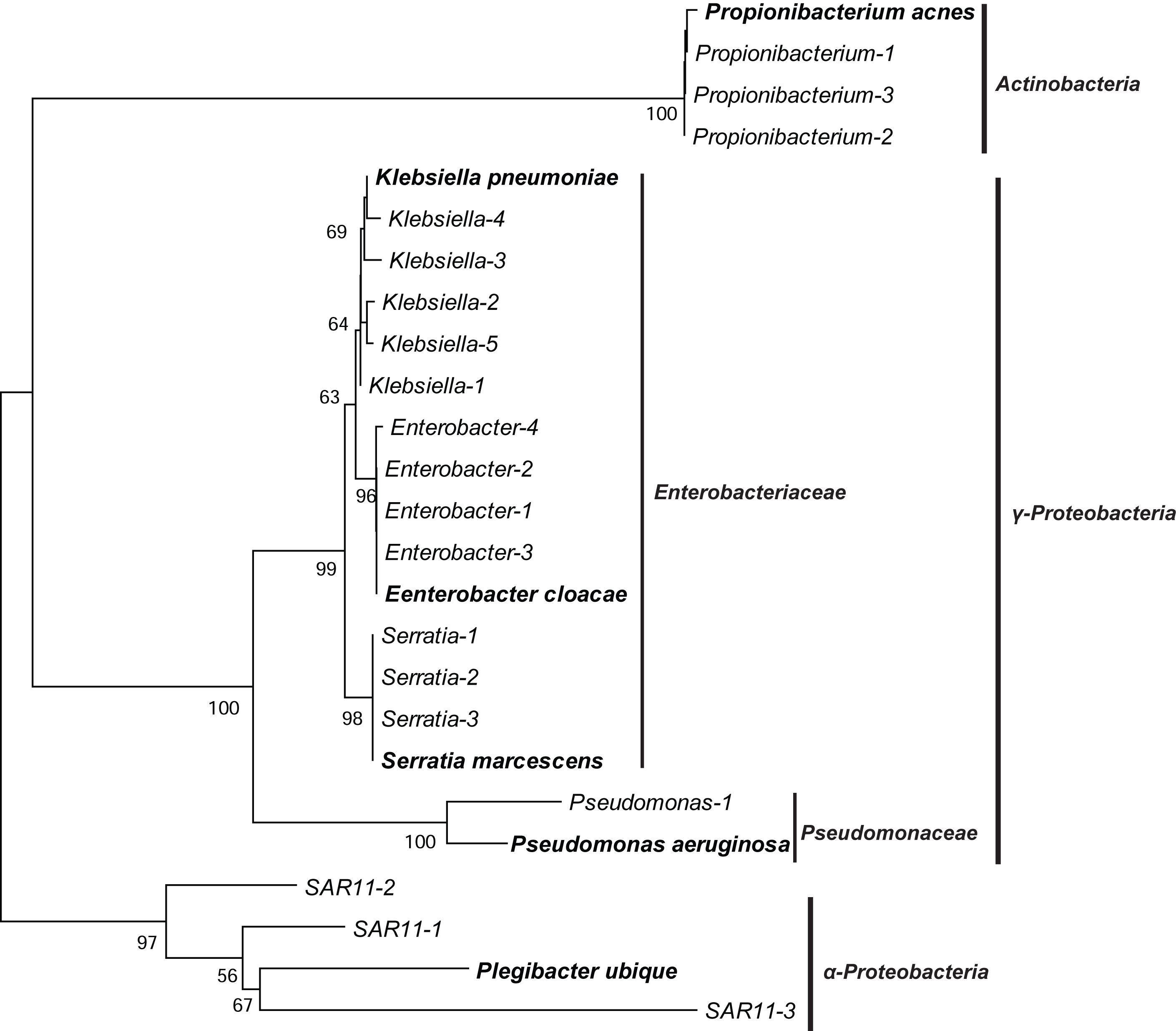

Supplement: Figure S3 — Phylogenetic relatedness of representative bacterial species and mosquito gut bacteria based on 16S rRNA tag sequence similarity. Neighbor-joining tree is presented. Bootstrap values (>50) are given at nodes. Representative bacteria are presented in bold fonts. (TIF) [file pone.0024767.s003.tif]
