# Supplementary material for: Dynamic Gut Microbiome across Life History of the Malaria Mosquito Anopheles gambiae in Kenya
Source: PLoS One. 2011 Sep 21;6(9):e24767. doi: 10.1371/journal.pone.0024767 (PMC3177825; doi:10.1371/journal.pone.0024767)
Supplement: Table S1 — Similarity-based OTUs and species richness estimates. (PDF) [file pone.0024767.s004.pdf]

Table S1. Similarity-based OTUs and species richness estimates

		Cluster distance								
		3%			5%			10%		
Sample	Reads	OTUs	ACE	Chao1	OTUs	ACE	Chao1	OTUs	ACE	Chao1
Habitat	19864	1831	9754	5400	1270	4584	2789	588	1714	1138
Larva	29152	2007	6454	4267	1226	3142	2344	542	1166	1035
Pupa	25713	1488	8171	4602	1048	4345	2715	489	1359	917
1-day-old adult, no sugar feeding	41881	855	4107	2273	579	1768	1170	294	570	426
3-day-old, sugar fed	34318	628	3497	1990	363	939	707	191	335	298
7-day-old, sugar fed	37554	506	2331	1409	291	847	625	156	219	212
2 days post blood meal	37416	277	1161	693	114	174	169	34	42	39
4 days post blood meal	28020	240	892	570	97	240	180	46	77	66
7 days post blood meal	38065	238	862	571	120	210	176	71	122	97

Cluster distance was calculated based on sequence difference against Silva reference alignment, and species richness estimates were calculated by using the program Mothur as described in Methods.
