# Supplementary material for: Dynamic Gut Microbiome across Life History of the Malaria Mosquito Anopheles gambiae in Kenya
Source: PLoS One. 2011 Sep 21;6(9):e24767. doi: 10.1371/journal.pone.0024767 (PMC3177825; doi:10.1371/journal.pone.0024767)
Supplement: Table S2 — Gut bacterial composition at phylum level across life stages of An.gambiae . (PDF) [file pone.0024767.s005.pdf]

Table S2. Gut bacterial composition at phylum level across life stages of *An.gambiae*

Phylum	Taxon abudance (%) and standard error (S.E.)																	
	Habitat		Larva		Pupa		1-day-old, no feeding		3-day-old, sugar fed		7-day-old, sugar fed		2 days post blood meal		4 days post blood meal		7 days post blood meal	
	%	S.E.	%	S.E.	%	S.E.	%	S.E.	%	S.E.	%	S.E.	%	S.E.	%	S.E.	%	S.E.
Actinobacteria	9.46	6.03	15.91	11.76	0.93	0.55	11.33	6.59	11.75	6.78	8.61	8.11	0.14	0.12	0.18	0.02	1.29	1.13
Bacteroidetes	4.11	1.31	4.38	1.29	2.42	0.77	1.89	0.42	13.82	6.07	62.32	22.75	3.73	3.59	67.93	14.84	84.19	5.22
Cyanobacteria	29.20	6.23	39.74	9.38	42.56	21.15	0.22	0.12	0.33	0.31	0.30	0.17	0.01	0.01	0.02	0.02	0.26	0.13
Firmicutes	8.69	3.19	4.57	1.98	0.60	0.25	5.34	1.52	2.65	1.31	1.29	0.27	0.01	0.01	0.09	0.04	1.39	1.36
Proteobacteria	41.22	3.94	26.12	3.48	45.69	26.34	78.54	6.23	70.52	5.96	18.52	8.41	96.02	3.60	31.68	14.85	12.66	5.73
Gemmatimonadetes	0.56	0.14	0.57	0.29	0.34	0.16	0.00	0.00	0.03	0.03	0.00	0.00	0.00	0.00	0.00	0.00	0.00	0.00
Planctomycetes	0.29	0.29	0.63	0.15	0.16	0.09	0.01	0.01	0.00	0.00	0.16	0.16	0.01	0.01	0.00	0.00	0.06	0.06
OP10	0.19	0.16	0.26	0.03	0.14	0.13	0.00	0.00	0.00	0.00	0.00	0.00	0.00	0.00	0.00	0.00	0.00	0.00
SR1	0.04	0.04	0.00	0.00	0.00	0.00	0.05	0.05	0.00	0.00	0.08	0.08	0.00	0.00	0.00	0.00	0.00	0.00
Verrucomicrobia	0.01	0.01	0.24	0.08	0.22	0.18	0.00	0.00	0.00	0.00	0.07	0.07	0.00	0.00	0.00	0.00	0.02	0.02
Acidobacteria	0.00	0.00	0.76	0.21	0.11	0.10	0.08	0.06	0.04	0.04	0.00	0.00	0.00	0.00	0.00	0.00	0.03	0.02
Bacteria incertae sedis	0.00	0.00	0.00	0.00	0.00	0.00	0.00	0.00	0.00	0.00	0.14	0.14	0.00	0.00	0.00	0.00	0.00	0.00
Chloroflexi	0.00	0.00	0.18	0.05	0.07	0.07	0.00	0.00	0.00	0.00	0.00	0.00	0.00	0.00	0.00	0.00	0.05	0.03
Deinococcus-Thermus	0.00	0.00	0.00	0.00	0.00	0.00	0.02	0.01	0.00	0.00	0.00	0.00	0.00	0.00	0.00	0.00	0.00	0.00
Fusobacteria	0.00	0.00	0.01	0.01	0.46	0.42	1.15	1.12	0.00	0.00	0.35	0.35	0.00	0.00	0.00	0.00	0.00	0.00
OD1	0.00	0.00	0.00	0.00	0.00	0.00	0.03	0.02	0.00	0.00	0.00	0.00	0.00	0.00	0.00	0.00	0.00	0.00
TM7	0.00	0.00	0.02	0.01	0.01	0.01	0.33	0.31	0.03	0.03	0.01	0.01	0.04	0.04	0.00	0.00	0.05	0.03
unclassified bactera	6.22	0.92	6.62	0.51	6.29	3.12	1.00	0.17	0.83	0.16	8.13	6.82	0.02	0.01	0.10	0.03	0.05	0.03
Total reads	23892		30025		31657		44218		50976		39336		113663		53789		38768	

The percentage value is presented as Mean of three replicates.
