# Supplementary material for: Dynamic Gut Microbiome across Life History of the Malaria Mosquito Anopheles gambiae in Kenya
Source: PLoS One. 2011 Sep 21;6(9):e24767. doi: 10.1371/journal.pone.0024767 (PMC3177825; doi:10.1371/journal.pone.0024767)
Supplement: Table S3 — Gut bacterial composition at family level across life stages of An.gambiae . (PDF) [file pone.0024767.s006.pdf]

Table S3. Gut bacterial composition at family level across life stages of *An.gambiae*

Family	Taxon abudance (%) and standard error (S.E.)																	
	Habitat		Larva		Pupa		1-day-old, no feeding		3-day-old, sugar fed		7-day-old, sugar fed		2 days post blood meal		4 days post blood meal		7 days post blood meal	
	%	S.E.	%	S.E.	%	S.E.	%	S.E.	%	S.E.	%	S.E.	%	S.E.	%	S.E.	%	S.E.
Chloroplast	18.31	4.63	33.73	8.90	38.00	19.01	0.10	0.09	0.33	0.30	0.30	0.17	0.01	0.01	0.02	0.02	0.26	0.13
Aeromonadaceae	0.06	0.06	0.80	0.78	31.23	31.21	0.10	0.06	1.23	1.22	0.00	0.00	4.19	2.93	0.33	0.20	1.54	0.38
Comamonadaceae	0.84	0.44	1.04	0.09	2.31	1.17	0.07	0.05	2.31	2.16	0.25	0.12	0.10	0.04	0.35	0.08	0.44	0.19
Erythrobacteraceae	1.35	0.77	4.54	2.00	1.68	0.87	0.00	0.00	0.00	0.00	0.00	0.00	0.00	0.00	0.00	0.00	0.00	0.00
Rhodobacteraceae	3.23	1.89	4.30	1.32	1.16	0.62	0.04	0.03	0.56	0.55	0.22	0.19	0.00	0.00	0.74	0.17	1.21	0.10
Enterobacteriaceae	0.08	0.05	4.71	0.75	1.00	0.72	69.38	10.05	37.42	20.24	5.51	4.76	86.58	3.93	21.45	11.11	5.77	5.46
Cyanobacteria/Family V	0.01	0.01	2.93	1.67	0.77	0.41	0.00	0.00	0.00	0.00	0.00	0.00	0.00	0.00	0.00	0.00	0.00	0.00
Sphingomonadaceae	3.50	1.65	0.72	0.37	0.38	0.15	0.03	0.03	6.85	5.82	1.87	1.77	0.02	0.02	0.08	0.02	0.29	0.11
Xanthomonadaceae	0.39	0.27	0.21	0.13	0.36	0.23	1.49	1.45	11.19	11.15	0.37	0.26	0.01	0.01	0.01	0.00	0.26	0.25
Flavobacteriaceae	1.44	1.43	0.77	0.20	0.31	0.20	0.78	0.45	13.24	6.15	61.71	23.33	3.72	3.59	67.92	14.84	84.17	5.21
Propionibacteriaceae	2.97	1.61	0.30	0.12	0.13	0.06	9.09	5.67	11.10	6.36	4.86	4.50	0.02	0.02	0.07	0.03	0.75	0.75
Pseudomonadaceae	0.27	0.10	0.15	0.12	0.08	0.06	0.14	0.12	1.78	1.78	1.14	0.98	4.03	2.00	6.09	4.42	0.54	0.34
Cyanobacteria/Family II	10.01	1.38	1.11	1.07	0.07	0.05	0.12	0.12	0.00	0.00	0.00	0.00	0.00	0.00	0.00	0.00	0.00	0.00
Acetobacteraceae	6.68	1.64	0.76	0.21	0.07	0.02	0.10	0.06	0.00	0.00	0.00	0.00	0.00	0.00	0.14	0.11	0.13	0.13
Microbacteriaceae	2.45	2.44	8.55	8.09	0.05	0.04	0.00	0.00	0.01	0.01	0.05	0.03	0.01	0.01	0.03	0.01	0.20	0.18
Moraxellaceae	0.16	0.16	0.07	0.04	0.05	0.01	0.25	0.24	1.25	0.94	4.02	2.75	0.92	0.28	1.79	0.71	1.26	0.56
Lachnospiraceae	0.10	0.10	0.12	0.07	0.01	0.01	1.37	1.32	0.00	0.00	0.06	0.06	0.00	0.00	0.00	0.00	0.27	0.27
Methylocystaceae	11.92	1.88	0.32	0.09	0.01	0.01	0.06	0.06	0.00	0.00	0.00	0.00	0.00	0.00	0.00	0.00	0.00	0.00
Aerococcaceae	2.16	2.16	0.00	0.00	0.00	0.00	0.01	0.01	0.00	0.00	0.02	0.02	0.00	0.00	0.00	0.00	0.00	0.00
Incertae Sedis XI	2.60	2.60	0.00	0.00	0.00	0.00	0.06	0.06	0.00	0.00	0.00	0.00	0.00	0.00	0.00	0.00	0.00	0.00
SAR11	0.01	0.00	0.03	0.03	0.00	0.00	0.07	0.06	5.61	5.60	3.83	3.81	0.00	0.00	0.00	0.00	0.00	0.00
Other	31.44	-	34.82	-	22.33	-	16.74	-	7.14	-	15.80	-	0.37	-	0.99	-	2.93	-

The percentage value is presented as Mean of three replicates. Taxa with abundance <2% in all samples and other taxa unssignable to a family were pooled together and are presented in "Other".
