# Supplementary material for: Dynamic Gut Microbiome across Life History of the Malaria Mosquito Anopheles gambiae in Kenya
Source: PLoS One. 2011 Sep 21;6(9):e24767. doi: 10.1371/journal.pone.0024767 (PMC3177825; doi:10.1371/journal.pone.0024767)
Supplement: Table S4 — Gut bacterial composition at genus level across life stages of An.gambiae . (PDF) [file pone.0024767.s007.pdf]

Table S4. Gut bacterial composition at genus level across life stages of *An.gambiae*

Taxon abudance (%) and standard error (S.E.)

Genus	Habitat		Larva		Pupa		1-day-old, no feeding		3-day-old, sugar fed		7-day-old, sugar fed		2 days post blood meal		4 days post blood meal		7 days post blood meal	
	%	S.E.	%	S.E.	%	S.E.	%	S.E.	%	S.E.	%	S.E.	%	S.E.	%	S.E.	%	S.E.
Klebsiella	0.00	0.00	0.00	0.00	0.00	0.00	0.20	0.19	3.78	3.78	0.00	0.00	22.60	5.85	0.21	0.15	4.48	4.38
Raoultella	0.00	0.00	0.00	0.00	0.00	0.00	0.56	0.56	10.90	10.89	0.00	0.00	16.01	15.08	1.88	1.88	0.26	0.23
Serratia	0.02	0.01	0.00	0.00	0.00	0.00	0.05	0.05	12.06	11.32	0.00	0.00	15.90	13.36	4.22	3.97	0.23	0.22
Enterobacter	0.00	0.00	0.01	0.01	0.07	0.07	0.12	0.12	2.02	2.02	2.48	2.40	12.32	3.09	4.73	4.24	0.34	0.29
Aeromonas	0.00	0.00	0.80	0.78	31.23	31.21	0.10	0.06	1.23	1.22	0.00	0.00	4.19	2.92	0.33	0.20	1.54	0.38
Pseudomonas	0.22	0.12	0.14	0.12	0.07	0.05	0.13	0.11	1.70	1.70	1.11	0.95	3.89	2.02	5.37	4.20	0.43	0.26
Elizabethkingia	0.03	0.02	0.00	0.00	0.14	0.12	0.24	0.23	5.58	4.83	61.56	23.38	3.70	3.58	67.85	14.86	84.10	5.21
Acinetobacter	0.00	0.00	0.04	0.03	0.03	0.02	0.25	0.23	1.24	0.93	4.00	2.75	0.91	0.26	1.79	0.71	1.25	0.55
Comamonas	0.00	0.00	0.00	0.00	0.00	0.00	0.00	0.00	2.19	2.19	0.06	0.03	0.08	0.02	0.07	0.05	0.11	0.05
Propionibacterium	2.94	1.70	0.18	0.09	0.09	0.05	9.08	5.67	11.09	6.35	4.76	4.40	0.01	0.01	0.07	0.03	0.75	0.75
Stenotrophomonas	0.28	0.16	0.00	0.00	0.10	0.10	1.24	1.20	10.97	10.92	0.37	0.26	0.01	0.01	0.01	0.00	0.25	0.25
Bacillariophyta	0.00	0.00	15.55	5.65	5.91	2.89	0.07	0.05	0.19	0.18	0.03	0.03	0.01	0.01	0.00	0.00	0.07	0.07
Thorsellia	0.01	0.01	3.58	0.42	0.26	0.25	67.63	9.07	2.55	2.45	0.01	0.01	0.01	0.01	0.00	0.00	0.00	0.00
Finegoldia	2.59	1.50	0.00	0.00	0.00	0.00	0.00	0.00	0.00	0.00	0.00	0.00	0.00	0.00	0.00	0.00	0.00	0.00
Chlorophyta	16.31	9.42	17.02	4.36	18.43	10.21	0.01	0.00	0.12	0.12	0.00	0.00	0.00	0.00	0.00	0.00	0.00	0.00
Methylocystis	11.55	6.67	0.30	0.09	0.01	0.01	0.05	0.05	0.00	0.00	0.00	0.00	0.00	0.00	0.00	0.00	0.00	0.00
GpIIa	10.01	5.78	1.11	1.07	0.07	0.05	0.12	0.12	0.00	0.00	0.00	0.00	0.00	0.00	0.00	0.00	0.00	0.00
Roseomonas	5.23	3.02	0.22	0.05	0.03	0.00	0.00	0.00	0.00	0.00	0.00	0.00	0.00	0.00	0.14	0.11	0.00	0.00
Novosphingobium	2.21	1.28	0.35	0.19	0.05	0.05	0.00	0.00	5.42	5.42	0.00	0.00	0.00	0.00	0.00	0.00	0.01	0.01
Aerococcus	2.15	1.24	0.00	0.00	0.00	0.00	0.00	0.00	0.00	0.00	0.00	0.00	0.00	0.00	0.00	0.00	0.00	0.00
Corynebacterium	1.46	0.84	0.07	0.06	0.04	0.03	1.16	1.03	0.00	0.00	0.28	0.28	0.00	0.00	0.00	0.00	0.00	0.00
Lactobacillus	1.46	0.84	0.00	0.00	0.20	0.14	0.26	0.26	1.58	1.17	0.04	0.04	0.00	0.00	0.00	0.00	0.00	0.00
Cloacibacterium	1.26	0.73	0.00	0.00	0.00	0.00	0.00	0.00	0.00	0.00	0.00	0.00	0.00	0.00	0.00	0.00	0.00	0.00
Rhizobium	1.21	0.70	1.03	0.24	0.04	0.03	0.00	0.00	0.01	0.01	0.00	0.00	0.00	0.00	0.04	0.02	0.02	0.02
Porphyrobacter	1.12	0.65	4.48	1.97	1.19	0.58	0.00	0.00	0.00	0.00	0.00	0.00	0.00	0.00	0.00	0.00	0.00	0.00
Agromyces	0.00	0.00	5.73	5.64	0.00	0.00	0.00	0.00	0.00	0.00	0.00	0.00	0.00	0.00	0.00	0.00	0.00	0.00
GpV	0.01	0.01	2.93	1.67	0.64	0.41	0.00	0.00	0.00	0.00	0.00	0.00	0.00	0.00	0.00	0.00	0.00	0.00
Clostridium	0.12	0.07	1.01	0.38	0.10	0.09	0.00	0.00	0.00	0.00	0.32	0.24	0.00	0.00	0.00	0.00	0.53	0.53
Hydrogenophaga	0.14	0.08	0.89	0.10	1.67	1.08	0.03	0.03	0.00	0.00	0.00	0.00	0.00	0.00	0.00	0.00	0.00	0.00
GpI	0.00	0.00	0.80	0.27	1.27	0.74	0.00	0.00	0.01	0.01	0.00	0.00	0.00	0.00	0.00	0.00	0.00	0.00
Methylophilus	0.25	0.14	0.68	0.20	1.11	0.15	0.00	0.00	0.00	0.00	0.00	0.00	0.00	0.00	0.00	0.00	0.00	0.00
Fusobacterium	0.00	0.00	0.01	0.01	0.44	0.42	1.14	1.12	0.00	0.00	0.35	0.35	0.00	0.00	0.00	0.00	0.00	0.00
Chryseobacterium	0.00	0.00	0.24	0.05	0.00	0.00	0.50	0.50	7.38	7.33	0.05	0.05	0.00	0.00	0.03	0.03	0.00	0.00
Pelagibacter	0.01	0.01	0.03	0.03	0.00	0.00	0.07	0.06	5.61	5.60	3.83	3.81	0.00	0.00	0.00	0.00	0.00	0.00
Sphingobium	0.00	0.00	0.00	0.00	0.00	0.00	0.03	0.03	0.38	0.30	1.37	1.37	0.00	0.00	0.01	0.01	0.01	0.01

The percentage value is presented as Mean of three replicates. Taxa with abundance >1% in at least one sample are presented.
