# Supplementary material for: Dynamic Gut Microbiome across Life History of the Malaria Mosquito Anopheles gambiae in Kenya
Source: PLoS One. 2011 Sep 21;6(9):e24767. doi: 10.1371/journal.pone.0024767 (PMC3177825; doi:10.1371/journal.pone.0024767)
Supplement: Table S5 — Differentially abundant families between pupal and newly emerged adult guts. (PDF) [file pone.0024767.s008.pdf]

Table S5. Differentially abundant families between pupal and newly emerged adult guts

Family	Pupa (triplicates)			1-day-old adult,no feeding (triplicates)			P value	Q value
	mean %	variance	std.err	mean %	variance	std.err		
Chloroplast	37.967	10.871	19.036	0.104	0.000	0.087	0	-0.00247
Aeromonadaceae	21.759	14.177	21.739	0.102	0.000	0.057	0	-0.00247
Comamonadaceae	2.294	0.042	1.180	0.069	0.000	0.049	1.80E-163	-0.00247
Erythrobacteraceae	1.679	0.023	0.872	0.000	0.000	0.000	2.21E-136	-0.00247
Rhodobacteraceae	1.149	0.012	0.631	0.042	0.000	0.031	1.08E-94	-0.00247
Enterobacteriaceae	0.761	0.007	0.488	69.380	3.028	10.047	0	-0.00247
Propionibacteriaceae	0.125	0.000	0.063	9.091	0.965	5.672	0	-0.00247

The differential abundant taxa between collections were detected with Metastats
