# Supplementary material for: Dynamic Gut Microbiome across Life History of the Malaria Mosquito Anopheles gambiae in Kenya
Source: PLoS One. 2011 Sep 21;6(9):e24767. doi: 10.1371/journal.pone.0024767 (PMC3177825; doi:10.1371/journal.pone.0024767)
Supplement: Table S6 — Differentially abundant families before and after a blood meal. (PDF) [file pone.0024767.s009.pdf]

Table S6. Differentially abundant families before and after a blood meal

Family	3-day-old,sugar (triplicates)			2 days post blood meal (triplicates)			P value	Q value
	mean %	variance	std.err	mean %	variance	std.err		
Enterobacteriaceae	38.58	12.90	20.73	86.69	0.46	3.91	0	0
Aeromonadaceae	1.26	0.05	1.25	4.20	0.26	2.93	4.35E-134	2.55E-138
Pseudomonadaceae	1.82	0.10	1.82	4.04	0.12	2.00	1.58E-36	8.56E-41
Moraxellaceae	1.30	0.03	0.99	0.92	0.00	0.28	8.00E-30	4.01E-34
Flavobacteriaceae	13.64	1.21	6.35	3.73	0.39	3.60	0	0
Comamonadaceae	2.39	0.15	2.23	0.10	0.00	0.04	0	0
SAR11	5.86	1.03	5.85	0.00	0.00	0.00	0	0
Xanthomonadaceae	11.58	3.99	11.53	0.01	0.00	0.01	0.00E+00	0.00E+00
Sphingomonadaceae	7.09	1.09	6.02	0.02	0.00	0.02	0.00E+00	0.00E+00
Propionibacteriaceae	11.56	1.32	6.64	0.02	0.00	0.02	0.00E+00	0.00E+00

The differential abundant taxa between collections were detected with Metastats
