# Supplementary material for: Dynamic Gut Microbiome across Life History of the Malaria Mosquito Anopheles gambiae in Kenya
Source: PLoS One. 2011 Sep 21;6(9):e24767. doi: 10.1371/journal.pone.0024767 (PMC3177825; doi:10.1371/journal.pone.0024767)
Supplement: Table S7 — Differentially abundant genera before and after a blood meal. (PDF) [file pone.0024767.s010.pdf]

Table S7. Differentially abundant genera before and after a blood meal

Genus	3-day-old,sugar fed (triplicates)			2 days post blood meal (triplicates)			P value	Q value
	mean %	variance	std.err	mean %	variance	std.err		
Raoultella	16.523	8.183	16.516	24.037	15.515	22.741	3.16E-32	5.31E-32
Enterobacter	3.056	0.280	3.056	27.903	6.364	14.565	2.20E-12	2.77E-12
Aeromonas	1.862	0.104	1.858	5.908	0.485	4.021	0.002298	0.002106
Pseudomonas	2.577	0.199	2.577	6.318	0.164	2.342	0.027209	0.022859
Elizabethkingia	8.288	1.643	7.402	11.283	3.696	11.099	0.000265	0.000267
Comamonas	2.455	0.181	2.455	0.170	0.000	0.089	6.08E-09	6.81E-09
Propionibacterium	12.781	1.647	7.408	0.017	0.000	0.017	2.07E-75	2.09E-74
Stenotrophomonas	12.332	4.511	12.263	0.031	0.000	0.024	7.15E-73	3.60E-72
Bacillariophyta	0.283	0.002	0.279	0.028	0.000	0.025	0.02991	0.023196
Thorsellia	2.994	0.245	2.855	0.011	0.000	0.011	1.52E-14	2.19E-14
Novosphingobium	6.090	1.113	6.090	0.000	0.000	0.000	1.54E-40	3.11E-40
Chryseobacterium	8.287	2.035	8.237	0.000	0.000	0.000	1.50E-55	5.03E-55
Pelagibacter	6.547	1.280	6.532	0.000	0.000	0.000	5.20E-43	1.31E-42

The differential abundant taxa between collections were detected with Metastats
