# Supplementary material for: Dynamic Gut Microbiome across Life History of the Malaria Mosquito Anopheles gambiae in Kenya
Source: PLoS One. 2011 Sep 21;6(9):e24767. doi: 10.1371/journal.pone.0024767 (PMC3177825; doi:10.1371/journal.pone.0024767)
Supplement: Table S8 — Genomic comparison on anti-stress subsystems in abundant bacteria species from blood fed and sugar fed guts. (PDF) [file pone.0024767.s011.pdf]

Table S8. Genomic comparison on anti-stress subsystems in abundant bacteria species from blood fed * and sugar fed guts**

Subsystem	Gene and Role	Presence in bacterial genome					
		*Pseudomonas aeruginosa**	*Enterobacter cloacae**	*Serratia marcescens**	*Klebsiella pneumonia**	*Pelagibacter ubique***	*Propionibacteria acnes***
Flavohaemoglobin	Anaerobic nitric oxide reductase flavorubredoxin	no	yes	no	yes	no	no
Oxidative stress	Ferric uptake regulation protein FUR	yes	yes	yes	yes	yes	no
Bacterial hemoglobins	Flavohemoprotein (Hemoglobin-like protein) (Flavohemoglobin) (Nitric oxide dioxygenase) (EC 1.14.12.17)	yes	yes	yes	yes	no	no
Oxidative stress	Fumarate and nitrate reduction regulatory protein	yes	yes	yes	yes	no	no
Flavohaemoglobin	Functional role page for Anaerobic nitric oxide reductase transcription regulator NorR	yes	yes	no	yes	no	no
Flavohaemoglobin	Nitric oxide reductase activation protein NorD	yes	no	no	no	no	no
Flavohaemoglobin	Nitric oxide reductase activation protein NorQ	yes	no	no	no	no	no
Flavohaemoglobin	Nitric oxide reductase FlRd-NAD(+) reductase (EC 1.18.1.-)	no	yes	no	yes	no	no
Flavohaemoglobin	Nitric oxide-dependent regulator DnrN or NorA	no	yes	yes	no	no	no
Oxidative stress	Nitrite-sensitive transcriptional repressor NsrR	no	yes	yes	yes	no	no
Flavohaemoglobin	Nitrogen regulatory protein P-II	yes	yes	yes	no	no	no
Flavohaemoglobin	Nitrous oxide reductase maturation protein NosF (ATPase)	yes	no	no	no	no	no
Flavohaemoglobin	Nitrous oxide reductase maturation protein, outer-membrane lipoprotein NosL	yes	no	no	no	no	no
Flavohaemoglobin	Nitrous oxide reductase maturation transmembrane protein NosY	yes	no	no	no	no	no
Oxidative stress	NnrS protein involved in response to NO	yes	no	no	no	no	no
Glutaredoxins	Oxygen-insensitive NADPH nitroreductase (EC 1.-.-.-)	no	yes	no	yes	no	no
Oxidative stress	transcriptional regulator, Crp/Fnr family	yes	no	no	no	yes	no
Oxidative stress	Catalase (EC 1.11.1.6)	yes	yes	yes	yes	yes	yes

Oxidative stress	Superoxide dismutase [Cu-Zn] precursor (EC 1.15.1.1)	no	yes	yes	yes	no	no
Oxidative stress	Superoxide dismutase [Fe] (EC 1.15.1.1)	yes	yes	yes	yes	no	no
Oxidative stress	Superoxide dismutase [Mn] (EC 1.15.1.1)	yes	yes	yes	yes	yes	yes
Oxidative stress	Hydrogen peroxide-inducible genes activator, OxyR	yes	yes	yes	yes	yes	no
Oxidative stress	Redox-sensitive transcriptional activator SoxR	yes	yes	yes	yes	no	no
Oxidative stress	Regulatory protein SoxS	no	yes	no	yes	no	no
Glutaredoxins	23S rRNA (Uracil-5-) -methyltransferase rumB (EC 2.1.1.-)	no	yes	yes	yes	no	no
Flavohaemoglobin	3-phenylpropionate dioxygenase ferredoxin subunit	no	yes	no	no	no	no
Flavohaemoglobin	ABC-type Fe3+-siderophore transport system, permease 2 component	no	no	yes	no	no	no
Glutathione analogs: mycothiol	Acetyl-CoA:Cys-GlcN-Ins acetyltransferase, mycothiol synthase MshD	no	no	no	no	no	no
Regulation of Oxidative Stress Response	Aerobic respiration control protein arcA	no	no	no	yes	no	no
Regulation of Oxidative Stress Response	Aerobic respiration control sensor protein arcB (EC 2.7.3.-)	no	no	no	yes	no	no
Oxidative stress	Alkyl hydroperoxide reductase subunit C-like protein	yes	yes	yes	yes	yes	no
Flavohaemoglobin	Attachment invasion locus protein precursor	no	no	yes	no	no	no
Oxidative stress	bacteriophytochrome heme oxygenase BphO	yes	no	no	no	no	no
Carbon Starvation	Carbon starvation protein A	yes	yes	no	no	no	yes
Carbon Starvation	Carbon starvation protein A paralog	no	yes	yes	no	no	no
Carbon Starvation	Carbon storage regulator	yes	yes	no	yes	no	no
Glutaredoxins	Cell division protein BolA	yes	yes	yes	no	no	no
Glutaredoxins	Cell wall endopeptidase, family M23/M37	no	yes	yes	yes	no	no
Glutaredoxins	Chaperone protein HscA	yes	yes	yes	no	no	no
Glutaredoxins	Chaperone protein HscB	yes	yes	yes	no	no	no
Glutaredoxins	Competence protein F homolog, phosphoribosyltransferase domain	no	yes	yes	no	no	no

Protection from Reactive Oxygen Species	Cytochrome c551 peroxidase (EC 1.11.1.5)	yes	no	no	no	yes	no
Flavohaemoglobin	Cytochrome O ubiquinol oxidase subunit I (EC 1.10.3.-)	yes	yes	yes	yes	no	no
Flavohaemoglobin	Cytochrome O ubiquinol oxidase subunit II (EC 1.10.3.-)	yes	yes	yes	yes	no	no
Flavohaemoglobin	Cytochrome O ubiquinol oxidase subunit III (EC 1.10.3.-)	yes	yes	yes	yes	no	no
Flavohaemoglobin	Cytochrome O ubiquinol oxidase subunit IV (EC 1.10.3.-)	yes	yes	yes	yes	no	no
Bacterial hemoglobins	diguanylate cyclase/phosphodiesterase (GGDEF & EAL domains) with PAS/PAC sensor(s)	yes	yes	no	no	no	no
Flavohaemoglobin	Electron transport protein HydN	no	yes	no	yes	no	no
Oxidative stress	Fe2+/Zn2+ uptake regulation proteins	yes	no	no	no	no	no
Flavohaemoglobin	Ferrichrome transport ATP-binding protein FhuC (TC 3.A.1.14.3)	no	no	no	no	no	no
Oxidative stress	Ferroxidase (EC 1.16.3.1)	yes	yes	yes	yes	yes	no
Glutathione: Non-redox reactions	FIG005121: SAM-dependent methyltransferase (EC 2.1.1.-)	yes	yes	yes	no	no	no
Glutaredoxins	FIG136845: Rhodanese-related sulfurtransferase	yes	yes	yes	no	no	no
Glutathione: Biosynthesis and gamma-glutamyl cycle	Gamma-glutamyltranspeptidase (EC 2.3.2.2)	yes	yes	yes	no	no	no
Glutathione: Biosynthesis and gamma-glutamyl cycle	Glutamate--cysteine ligase (EC 6.3.2.2)	yes	yes	yes	yes	no	no
Glutathione: Redox cycle	Glutaredoxin	yes	no	no	no	no	no
Glutathione: Redox cycle	Glutaredoxin 1	no	yes	yes	yes	no	no
Glutathione: Redox cycle	Glutaredoxin 2	no	no	yes	no	no	no
Glutathione: Redox cycle	Glutaredoxin 3 (Grx3)	yes	yes	yes	no	no	no
Glutathione: Redox cycle	Glutaredoxin-like protein NrdH, required for reduction of Ribonucleotide reductase class Ib	no	yes	yes	no	no	no

Glutathione: Redox cycle	Glutaredoxin-related protein	yes	no	yes	no	no	no
Glutathione: Redox cycle	Glutathione peroxidase (EC 1.11.1.9)	yes	yes	yes	yes	yes	no
Glutathione: Redox cycle	Glutathione peroxidase family protein	yes	no	yes	yes	no	no
Glutathione: Redox cycle	Glutathione reductase (EC 1.8.1.7)	yes	yes	yes	yes	no	no
Glutathione: Non-redox reactions	Glutathione S-transferase (EC 2.5.1.18)	yes	yes	yes	yes	no	no
Glutathione: Non-redox reactions	Glutathione S-transferase family protein	yes	no	no	no	no	no
Glutathione: Non-redox reactions	Glutathione S-transferase, omega (EC 2.5.1.18)	yes	yes	yes	no	no	no
Glutathione: Non-redox reactions	Glutathione S-transferase, theta (EC 2.5.1.18)	no	no	no	yes	no	no
Glutathione: Non-redox reactions	Glutathione S-transferase, unnamed subgroup (EC 2.5.1.18)	yes	no	yes	no	no	no
Glutathione: Non-redox reactions	Glutathione S-transferase, unnamed subgroup 2 (EC 2.5.1.18)	yes	no	no	no	no	no
Glutathione: Non-redox reactions	Glutathione S-transferase, zeta (EC 2.5.1.18)	yes	no	yes	no	no	no
Glutaredoxins	Glutathione synthetase (EC 6.3.2.3)	yes	yes	yes	no	no	no
Glutathionylspermidine and Trypanothione	Glutathionylspermidine amidohydrolase (EC 3.5.1.78)	no	yes	no	no	no	no
Glutathionylspermidine and Trypanothione	Glutathionylspermidine synthase (EC 6.3.1.8)	no	yes	no	no	no	no
Glutaredoxins	Glycerol-3-phosphate dehydrogenase [NAD(P)+] (EC 1.1.1.94)	yes	yes	yes	no	no	no
Glutathione analogs: mycothiol	Glycosyltransferase MshA involved in mycothiol biosynthesis (EC 2.4.1.-)	no	no	no	no	no	no
Hfl operon	GTP-binding protein HflX	yes	yes	yes	yes	yes	yes
Flavohaemoglobin	H(+)/Cl(-) exchange transporter ClcA	no	yes	yes	yes	no	no
Flavohaemoglobin	Heme O synthase, protoheme IX farnesyltransferase (EC 2.5.1.-) COX10-CtaB	yes	yes	yes	no	no	no
Oxidative stress	Heme oxygenase HemO, associated with heme uptake	yes	no	no	no	no	no
Hfl operon	HflC protein	yes	yes	yes	yes	no	no
Hfl operon	HflK protein	yes	yes	yes	yes	no	no

Glutathione: Non-redox reactions	Hydroxyacylglutathione hydrolase (EC 3.1.2.6)	yes	yes	yes	yes	yes	no
Glutathione: Non-redox reactions	Hypothetical metal-binding enzyme, YcbL homolog	yes	yes	yes	no	no	no
Flavohaemoglobin	Inositol-1-monophosphatase (EC 3.1.3.25)	yes	yes	yes	yes	no	no
Flavohaemoglobin	Iron binding protein IscA for iron-sulfur cluster assembly	yes	yes	yes	no	no	no
Flavohaemoglobin	Iron(III) dicitrate transport ATP-binding protein FecE (TC 3.A.1.14.1)	no	no	yes	no	no	no
Flavohaemoglobin	Iron(III) dicitrate transport system permease protein FecD (TC 3.A.1.14.1)	no	no	yes	yes	no	no
Oxidative stress	Iron-binding ferritin-like antioxidant protein	yes	yes	yes	yes	yes	no
Flavohaemoglobin	Iron-sulfur cluster assembly scaffold protein IscU	yes	yes	yes	no	no	no
Flavohaemoglobin	Iron-sulfur cluster regulator IscR	yes	yes	yes	no	no	no
Flavohaemoglobin	Iron-sulfur cluster-binding protein, Rieske family	no	no	no	no	no	no
Glutathione: Non-redox reactions	Lactoylglutathione lyase (EC 4.4.1.5)	yes	yes	yes	yes	yes	no
Glutathione analogs: mycothiol	L-cysteine:1D-myo-inositYl 2-amino-2-deoxy-alpha-D-glucopyranoside ligase MshC	no	no	no	no	no	no
Flavohaemoglobin	Macrophage infectivity potentiator	no	no	no	no	no	no
Glutathione: Biosynthesis and gamma-glutamyl cycle	Membrane alanine aminopeptidase N (EC 3.4.11.2)	yes	yes	yes	no	no	no
Flavohaemoglobin	Membrane protein, suppressor for copper-sensitivity ScsD	no	yes	yes	yes	no	no
Oxidative stress	Metallothionein	yes	no	no	no	no	no
Glutathione: Non-redox reactions	Methylglyoxal synthase (EC 4.2.3.3)	no	yes	yes	yes	yes	no
Commensurate regulon activation	Multiple antibiotic resistance protein MarA	no	yes	no	yes	no	no
Glutathione analogs: mycothiol	Mycothiol S-conjugate amidase Mca	no	no	no	no	no	no
Glutathione analogs: mycothiol	N-acetyl-1-D-myo-inositYl-2-amino-2-deoxy-alpha-D-glucopyranoside deacetylase MshB	no	no	no	no	no	no

Flavohaemoglobin	NAD synthetase (EC 6.3.1.5) / Glutamine amidotransferase chain of NAD synthetase	no	no	yes	no	no	no
Redox-dependent regulation of nucleus processes	NAD-dependent glyceraldehyde-3-phosphate dehydrogenase (EC 1.2.1.12)	yes	yes	yes	yes	yes	yes
Redox-dependent regulation of nucleus processes	NAD-dependent protein deacetylase of SIR2 family	yes	yes	yes	yes	yes	yes
NADPH:quinone oxidoreductase 2	NADPH:quinone oxidoreductase 2	no	yes	no	yes	no	no
Redox-dependent regulation of nucleus processes	NADPH-dependent glyceraldehyde-3-phosphate dehydrogenase (EC 1.2.1.13)	yes	no	no	no	no	no
Dimethylarginine metabolism	NG,NG-dimethylarginine dimethylaminohydrolase 1 (EC 3.5.3.18)	yes	no	no	no	yes	no
Redox-dependent regulation of nucleus processes	Nicotinamidase (EC 3.5.1.19)	yes	yes	yes	yes	yes	yes
Redox-dependent regulation of nucleus processes	Nicotinate phosphoribosyltransferase (EC 2.4.2.11)	yes	yes	yes	yes	yes	yes
Oxidative stress	Non-specific DNA-binding protein Dps	yes	yes	yes	yes	yes	no
Oxidative stress	Organic hydroperoxide resistance protein	yes	yes	yes	no	no	yes
Oxidative stress	Organic hydroperoxide resistance transcriptional regulator	yes	yes	yes	no	no	yes
Dimethylarginine metabolism	Ornithine aminotransferase (EC 2.6.1.13)	no	no	no	no	no	no
Oxidative stress	Paraquat-inducible protein A	yes	yes	yes	no	no	no
Oxidative stress	Paraquat-inducible protein B	yes	yes	yes	no	no	no
Glutaredoxins	Peptide chain release factor 1	yes	yes	yes	no	no	no
Flavohaemoglobin	Periplasmic thiol:disulfide oxidoreductase DsbB, required for DsbA reoxidation	yes	yes	yes	no	no	no
Oxidative stress	Peroxidase (EC 1.11.1.7)	no	yes	yes	yes	yes	no
Phage shock protein (psp) operon	Phage shock protein A	no	yes	yes	yes	no	no
Phage shock protein (psp) operon	Phage shock protein B	no	yes	yes	yes	no	no

Phage shock protein (psp) operon	Phage shock protein C	no	yes	yes	yes	no	no
Phage shock protein (psp) operon	Phage shock protein D	no	yes	yes	yes	no	no
Glutaredoxins	Phosphocarrier protein, nitrogen regulation associated	yes	yes	yes	yes	no	no
Oxidative stress	Phytochrome, two-component sensor histidine kinase (EC 2.7.3.-)	yes	no	no	no	no	no
Flavohaemoglobin	Probable 3-phenylpropionic acid transporter	yes	yes	yes	no	no	no
Glutathione: Non-redox reactions	Probable glutathione S-transferase (EC 2.5.1.18), YfcF homolog	no	yes	yes	no	no	no
Glutathione: Non-redox reactions	Probable glutathione S-transferase (EC 2.5.1.18), YfcG homolog	no	yes	yes	no	no	no
Glutaredoxins	Probable monothiol glutaredoxin GrlA	no	yes	no	no	no	no
Glutaredoxins	Protein export cytoplasm chaperone protein (SecB, maintains protein to be exported in unfolded state)	yes	yes	yes	no	no	no
Phage shock protein (psp) operon	Psp operon transcriptional activator	no	yes	yes	yes	no	no
Hfl operon	Putative inner membrane protein YjeT (clustered with HflC)	yes	yes	yes	yes	no	no
NADPH:quinone oxidoreductase 2	Redox-sensing transcriptional regulator QorR	no	yes	no	yes	no	no
Glutaredoxins	Rhodanese-like domain protein	yes	no	no	no	no	no
Glutaredoxins	Riboflavin kinase (EC 2.7.1.26)	yes	yes	yes	yes	no	no
Glutathione: Redox cycle	Ribonucleotide reductase of class Ib (aerobic), alpha subunit (EC 1.17.4.1)	no	yes	yes	yes	no	no
Commensurate regulon activation	Right origin-binding protein	no	yes	yes	yes	no	no
Flavohaemoglobin	RNA methyltransferase, TrmH family, group 1	no	yes	yes	yes	no	no
Hfl operon	RNA-binding protein Hfq	yes	yes	yes	yes	no	no
Rubrerythrin	Rubredoxin	yes	no	no	no	no	no
Rubrerythrin	Rubredoxin-NAD(+) reductase (EC 1.18.1.1)	yes	no	no	no	no	no
Flavohaemoglobin	Serine hydroxymethyltransferase (EC 2.1.2.1)	yes	yes	yes	no	no	no
Sugar-phosphate stress regulation	SgrR, sugar-phosphate stress, transcriptional activator of SgrS small RNA	no	yes	no	no	no	no

Glutathionylspermidine and Trypanothione	Similarity with glutathionylspermidine synthase (EC 6.3.1.8), group 1	no	yes	yes	no	no	no
Carbon Starvation	Starvation lipoprotein Slp paralog	no	yes	yes	yes	no	no
Carbon Starvation	Starvation sensing protein RspA	no	yes	yes	no	no	no
Carbon Starvation	Starvation sensing protein RspB	no	yes	yes	yes	no	no
Flavohaemoglobin	Stationary phase inducible protein CsiE	no	yes	yes	yes	no	no
Carbon Starvation	Stringent starvation protein A	yes	yes	yes	yes	no	no
Carbon Starvation	Stringent starvation protein B	yes	yes	yes	yes	no	no
Commensurate regulon activation	Transcriptional activator RamA	no	yes	no	yes	no	no
Glutathione: Biosynthesis and gamma-glutamyl cycle	Tripeptide aminopeptidase (EC 3.4.11.4)	no	yes	yes	no	no	no
Glutaredoxins	TrkA, Potassium channel-family protein	no	yes	yes	yes	no	no
Glutaredoxins	tRNA (cytosine34-2'-O-)-methyltransferase (EC 2.1.1.-)	yes	yes	yes	no	no	no
Flavohaemoglobin	tRNA:Cm32/Um32 methyltransferase	yes	yes	yes	yes	no	no
Glutathione: Non-redox reactions	Uncharacterized glutathione S-transferase-like protein	yes	yes	yes	no	no	no
Glutathione: Non-redox reactions	Uncharacterized GST-like protein yghU associated with glutathionylspermidine synthetase/amidase	no	yes	no	no	no	no
Glutathione: Non-redox reactions	Uncharacterized GST-like protein yibF	no	yes	yes	no	no	no
Glutathione: Non-redox reactions	Uncharacterized GST-like protein yncG	no	yes	yes	no	no	no
Universal stress protein family	Universal stress protein A	no	no	no	yes	no	no
Universal stress protein family	Universal stress protein B	no	no	no	yes	no	no
Universal stress protein family	Universal stress protein C	no	no	no	yes	no	no
Universal stress protein family	Universal stress protein E	no	no	no	yes	no	no
Universal stress protein family	Universal stress protein F	no	no	no	yes	no	no
Universal stress protein family	Universal stress protein family	yes	no	no	no	yes	no

Universal stress protein family	Universal stress protein family 4	no	no	no	no	no	no
Universal stress protein family	Universal stress protein family 5	yes	no	no	no	no	no
Universal stress protein family	Universal stress protein family 7	yes	no	no	no	no	no
Universal stress protein family	Universal stress protein G	no	no	no	yes	no	no
Oxidative stress	Zinc uptake regulation protein ZUR	yes	yes	yes	yes	yes	no
	Total # of genes that are present	98	117	108	81	23	10
