# Supplementary material for: Dynamic Gut Microbiome across Life History of the Malaria Mosquito Anopheles gambiae in Kenya
Source: PLoS One. 2011 Sep 21;6(9):e24767. doi: 10.1371/journal.pone.0024767 (PMC3177825; doi:10.1371/journal.pone.0024767)
Supplement: Table S9 — Comparison of gut bacterial composition at family level between Kenyan and lab samples. (PDF) [file pone.0024767.s012.pdf]

Table S9. Comparison of gut bacterial composition at family level between Kenyan and lab samples

Family	Taxon abundance %															
	Habitat		Larva		Pupa		1-day-old, no feeding		3-day-old, sugar fed		7-day-old, sugar fed		2 days PBM		4 days PBM	
	Lab	Kenya	Lab	Kenya	Lab	Kenya	Lab	Kenya	Lab	Kenya	Lab	Kenya	Lab	Kenya	Lab	Kenya
Chloroplast	0.00	18.31	0.01	16.64	0.00	24.14	0.01	0.10	0.02	0.32	0.00	0.29	0.01	0.00	0.00	0.01
Methylocystaceae	0.00	11.92	0.00	0.74	0.00	0.08	0.00	0.59	0.00	0.00	0.00	0.00	0.00	0.00	0.00	0.00
Family II	0.00	10.01	0.00	0.43	0.00	0.07	0.00	0.12	0.00	0.00	0.00	0.00	0.00	0.00	0.00	0.00
Acetobacteraceae	0.08	6.68	0.00	0.95	0.01	0.24	0.03	0.00	31.01	0.00	6.16	0.00	15.30	0.00	16.93	0.11
Sphingomonadaceae	6.57	3.50	0.06	1.06	0.67	0.53	0.10	0.06	0.03	12.82	0.00	1.76	0.00	0.04	0.00	0.50
Rhodobacteraceae	0.00	3.23	0.00	5.11	0.00	1.68	0.00	0.05	0.00	0.46	0.00	1.15	0.00	0.00	0.01	0.61
Propionibacteriaceae	0.00	2.97	0.00	0.22	0.00	0.11	0.25	7.51	0.00	7.71	0.02	4.18	0.00	0.02	0.00	0.06
Incertae Sedis XI	0.00	2.60	0.01	0.00	0.04	0.00	1.40	0.05	3.07	0.00	0.00	0.00	0.01	0.00	0.11	0.00
Microbacteriaceae	5.38	2.45	33.64	18.87	9.23	0.08	8.03	0.04	0.17	0.10	0.71	0.41	0.00	0.02	0.01	0.14
Aerococcaceae	0.00	2.16	0.00	0.00	0.00	0.00	0.15	0.01	0.00	0.00	0.00	0.02	0.00	0.00	0.00	0.00
Corynebacteriaceae	0.00	1.53	0.03	0.05	0.01	0.11	2.39	0.98	0.11	0.00	0.00	0.25	0.02	0.00	0.07	0.01
Lactobacillaceae	0.00	1.47	0.10	0.00	0.00	0.15	0.00	0.22	0.05	1.08	0.00	0.04	0.01	0.00	0.00	0.00
Flavobacteriaceae	17.76	1.44	4.05	1.06	53.02	0.29	57.14	0.77	54.80	14.34	92.53	54.77	39.78	1.76	57.51	56.42
Erythrobacteraceae	0.00	1.35	0.00	2.40	0.01	1.39	0.00	0.00	0.00	0.00	0.00	0.00	0.00	0.00	0.00	0.00
Rhizobiaceae	1.64	1.23	0.18	0.69	2.22	0.23	0.00	0.00	0.00	1.36	0.00	0.15	0.00	0.02	0.00	0.23
Chitinophagaceae	1.48	1.03	0.04	0.09	1.22	0.04	0.00	0.01	0.00	0.00	0.00	0.00	0.00	0.00	0.00	0.00
Hyphomicrobiaceae	0.00	1.03	0.00	0.40	0.00	0.54	0.00	0.00	0.00	0.02	0.04	0.00	0.00	0.00	0.00	0.00
Comamonadaceae	10.15	0.84	1.93	0.53	4.00	1.82	0.44	0.28	0.02	2.26	0.00	0.53	1.80	0.07	0.00	0.29
Nocardioidaceae	0.01	0.77	0.01	0.37	0.01	0.11	0.00	0.02	0.00	0.00	0.00	0.00	0.00	0.05	0.00	0.05
Intrasporangiaceae	0.00	0.60	0.00	0.09	0.00	0.00	0.00	0.00	0.14	0.01	0.00	0.02	0.00	0.00	0.00	0.00
Caulobacteraceae	0.07	0.60	0.25	0.26	2.26	0.59	0.00	0.01	0.00	0.00	0.00	0.34	0.00	0.00	0.00	0.15
Planococcaceae	0.00	0.59	0.00	0.00	0.00	0.00	0.00	0.00	0.00	0.00	0.00	0.00	0.00	0.00	0.00	0.00
Gemmatimonadaceae	0.00	0.56	0.00	0.28	0.00	0.22	0.00	0.00	0.00	0.02	0.00	0.00	0.00	0.00	0.00	0.00
Methylobacteriaceae	0.05	0.53	0.03	0.41	0.01	0.07	0.00	0.06	0.00	0.00	0.02	0.03	0.00	0.01	0.00	0.01
Xanthomonadaceae	0.00	0.39	0.07	0.69	0.40	0.90	0.21	1.55	0.14	8.58	0.02	0.35	0.00	0.01	0.00	0.01
Methylophilaceae	0.01	0.31	0.00	0.67	0.18	1.29	0.00	0.01	0.02	0.00	0.00	0.01	0.00	0.00	0.00	0.00
Micrococcaceae	0.00	0.31	0.00	0.06	0.00	0.05	0.54	0.09	0.00	0.04	0.00	0.02	0.00	0.00	0.00	0.05
Planctomycetaceae	0.00	0.29	0.02	0.31	0.00	0.11	0.00	0.01	0.00	0.00	0.00	0.14	0.00	0.01	0.00	0.00
Pseudomonadaceae	0.00	0.27	0.09	0.09	0.42	0.06	1.94	0.17	4.84	1.66	0.00	1.81	20.25	4.38	2.14	5.59
Staphylococcaceae	0.00	0.27	0.09	0.03	0.17	0.04	8.29	0.45	2.19	0.68	0.00	0.21	0.37	0.00	0.12	0.03
Prevotellaceae	0.00	0.26	0.00	0.00	0.00	0.02	0.15	0.10	0.06	0.00	0.00	0.07	0.00	0.00	0.00	0.00
Thermoactinomycetaceae	0.00	0.26	0.00	0.20	0.00	0.00	0.00	0.00	0.00	0.00	0.00	0.00	0.00	0.00	0.00	0.00
Clostridiaceae	3.18	0.24	0.50	1.14	0.48	0.22	0.03	0.02	0.00	0.00	0.00	0.71	0.00	0.00	0.00	0.00
Cytophagaceae	0.02	0.23	0.04	0.62	1.79	0.41	0.00	0.17	0.02	0.00	0.00	0.00	0.00	0.00	0.00	0.00
Rhodocyclaceae	0.67	0.17	0.11	0.33	0.12	0.11	0.00	0.01	0.00	0.00	0.00	0.01	0.00	0.00	0.00	0.00
Peptococcaceae	0.00	0.17	0.00	0.08	0.00	0.00	0.00	0.00	0.00	0.00	0.00	0.00	0.00	0.00	0.00	0.00
Moraxellaceae	0.01	0.16	0.19	0.04	1.52	0.08	0.66	0.24	0.03	1.18	0.02	3.97	0.00	0.61	0.00	1.59
Oxalobacteraceae	0.52	0.15	0.01	0.01	0.07	0.20	0.03	0.11	0.03	0.69	0.00	0.07	0.00	0.01	0.00	0.00

Sphingobacteriaceae	7.30	0.14	0.29	0.02	4.60	0.05	0.00	0.04	0.00	0.00	0.00	0.01	0.00	0.00	0.00	0.00
Lachnospiraceae	0.00	0.10	0.04	0.08	0.01	0.01	0.49	1.32	0.00	0.00	0.00	0.06	0.00	0.00	0.00	0.00
Veillonellaceae	0.01	0.09	0.00	0.05	0.00	0.01	0.05	1.26	0.00	0.01	0.00	0.10	0.00	0.00	0.00	0.00
Enterobacteriaceae	0.43	0.08	54.64	2.27	11.31	1.20	8.55	67.75	1.22	35.65	0.04	9.84	20.84	88.94	22.96	32.14
Burkholderiales_incertae_sedis	0.05	0.08	0.00	1.44	0.09	3.45	0.24	0.00	0.02	0.00	0.05	0.00	0.00	0.00	0.00	0.00
Paenibacillaceae	0.15	0.07	0.00	0.07	0.04	0.00	0.00	0.00	0.00	0.00	0.00	0.00	0.00	0.00	0.00	0.00
Aeromonadaceae	0.12	0.06	0.19	0.28	0.52	29.93	1.30	0.13	0.00	0.89	0.00	0.00	0.00	3.53	0.00	0.18
Geobacteraceae	0.00	0.06	0.00	0.01	0.00	0.00	0.00	0.00	0.00	0.00	0.00	0.00	0.00	0.00	0.00	0.00
Streptococcaceae	0.00	0.06	0.02	0.01	0.00	0.00	0.18	0.39	0.02	0.27	0.05	0.08	0.01	0.00	0.00	0.03
Ruminococcaceae	0.00	0.05	0.00	0.11	0.00	0.00	0.04	0.05	0.03	0.00	0.00	0.10	0.00	0.00	0.00	0.00
Alcaligenaceae	0.00	0.05	0.00	2.26	0.00	0.23	0.07	0.03	0.00	0.00	0.00	0.00	0.00	0.00	0.00	0.01
Cyclobacteriaceae	0.00	0.04	0.00	0.13	0.00	0.27	0.00	0.04	0.00	0.00	0.00	0.48	0.00	0.00	0.00	0.00
Burkholderiaceae	0.62	0.03	0.00	0.02	0.01	0.22	1.19	0.05	0.29	0.05	0.21	0.13	0.00	0.03	0.00	0.15
Eubacteriaceae	0.00	0.03	0.00	0.00	0.00	0.00	0.00	0.00	0.00	0.00	0.00	0.00	0.00	0.00	0.00	0.00
Nocardiaceae	0.00	0.03	0.00	0.03	0.00	0.00	0.00	0.00	0.00	0.00	0.02	0.00	0.00	0.00	0.00	0.00
Iamiaceae	0.00	0.02	0.00	0.06	0.04	0.00	0.00	0.00	0.00	0.00	0.00	0.00	0.00	0.00	0.00	0.00
Brucellaceae	0.00	0.02	0.00	0.01	0.00	0.00	0.00	0.00	0.00	0.00	0.00	0.00	0.00	0.00	0.00	0.00
Enterococcaceae	0.00	0.02	0.01	0.10	0.00	0.00	0.84	0.01	0.17	0.00	0.00	0.00	0.01	0.00	0.01	0.00
Rickettsiaceae	0.00	0.02	0.00	0.00	0.00	0.00	0.00	0.00	0.00	0.00	0.00	0.00	0.00	0.00	0.00	0.00
SAR11	0.00	0.01	0.00	0.01	0.00	0.00	0.00	0.07	0.00	3.94	0.00	3.36	0.00	0.00	0.00	0.00
Bradyrhizobiaceae	1.55	0.01	0.66	0.45	0.28	0.56	0.01	0.21	0.12	0.01	0.00	0.30	0.00	0.00	0.00	0.00
Family V	0.00	0.01	0.00	14.21	0.00	2.06	0.00	0.00	0.00	0.00	0.00	0.00	0.00	0.00	0.00	0.00
Verrucomicrobiaceae	0.00	0.01	0.00	0.00	0.00	0.00	0.00	0.00	0.00	0.00	0.00	0.00	0.00	0.00	0.00	0.00
Bdellovibrionaceae	0.00	0.00	0.00	0.02	0.00	0.01	0.00	0.00	0.00	0.00	0.00	0.00	0.00	0.00	0.00	0.00
Rhodospirillaceae	5.95	0.00	0.00	0.18	0.71	0.33	0.00	0.00	0.00	0.00	0.00	0.00	0.00	0.01	0.00	0.07
Bacteriovoracaceae	0.01	0.00	0.00	0.00	0.58	0.00	0.00	0.06	0.00	0.00	0.00	0.00	0.00	0.00	0.00	0.00
Neisseriaceae	27.66	0.00	0.00	0.06	0.00	0.42	0.00	0.01	0.00	0.00	0.00	0.00	0.00	0.00	0.00	0.00
Phyllobacteriaceae	0.07	0.00	0.00	0.08	0.00	0.01	0.00	0.06	0.00	0.00	0.00	0.00	0.00	0.00	0.00	0.04
Beijerinckiaceae	0.03	0.00	0.00	0.09	0.00	0.09	0.00	0.11	0.00	0.00	0.00	0.00	0.00	0.00	0.00	0.00
Family I	0.00	0.00	0.00	2.47	0.00	8.64	0.00	0.00	0.00	0.01	0.00	0.00	0.00	0.00	0.00	0.00
Family IV	0.00	0.00	0.00	1.34	0.00	0.10	0.00	0.00	0.00	0.00	0.00	0.00	0.00	0.00	0.00	0.00
Bacillaceae	0.00	0.00	0.01	0.64	0.00	0.03	0.00	0.00	0.03	0.00	0.00	0.07	0.00	0.00	0.00	0.01
Porphyromonadaceae	0.00	0.00	0.00	0.39	0.00	0.26	0.67	0.08	0.00	0.00	0.00	0.01	0.02	0.00	0.00	0.00
Micromonosporaceae	0.00	0.00	0.00	0.23	0.00	0.07	0.00	0.00	0.00	0.00	0.00	0.00	0.00	0.02	0.00	0.01
Mycobacteriaceae	0.00	0.00	0.01	0.15	0.00	0.01	0.00	0.00	0.00	0.00	0.00	0.00	0.00	0.00	0.00	0.00
Rubrobacteraceae	0.00	0.00	0.00	0.13	0.00	0.00	0.00	0.00	0.00	0.00	0.00	0.00	0.00	0.00	0.00	0.01
Geodermatophilaceae	0.00	0.00	0.00	0.12	0.00	0.00	0.00	0.18	0.00	0.00	0.00	0.00	0.00	0.00	0.00	0.00
Opitutaceae	0.00	0.00	0.00	0.12	0.00	0.15	0.00	0.00	0.08	0.00	0.00	0.06	0.00	0.00	0.00	0.00
Cryomorphaceae	0.00	0.00	0.11	0.10	0.00	0.09	0.00	0.09	0.06	0.00	0.00	0.09	0.00	0.00	0.00	0.00
Solirubrobacteraceae	0.00	0.00	0.00	0.10	0.00	0.07	0.00	0.00	0.00	0.00	0.00	0.00	0.00	0.00	0.00	0.00
Promicromonosporaceae	0.00	0.00	0.01	0.09	0.00	0.00	0.00	0.00	0.00	0.00	0.00	0.00	0.00	0.00	0.00	0.00
Peptostreptococcaceae	0.00	0.00	0.00	0.08	0.00	0.00	0.00	0.00	0.00	0.00	0.00	0.00	0.00	0.00	0.00	0.00
Pseudonocardiaceae	0.00	0.00	0.00	0.06	0.00	0.01	0.00	0.00	0.00	0.00	0.00	0.00	0.00	0.01	0.00	0.00

Streptomycetaceae	0.00	0.00	0.00	0.05	0.00	0.00	0.00	0.00	0.00	0.00	0.00	0.00	0.00	0.00	0.00	0.00
Chloroflexaceae	0.00	0.00	0.00	0.04	0.00	0.00	0.00	0.00	0.00	0.00	0.00	0.00	0.00	0.00	0.00	0.00
Saprospiraceae	0.00	0.00	0.00	0.04	0.00	0.02	0.00	0.14	0.00	0.00	0.00	0.00	0.00	0.00	0.00	0.00
Cryptosporangiaceae	0.00	0.00	0.00	0.03	0.00	0.02	0.00	0.00	0.00	0.00	0.00	0.00	0.00	0.00	0.00	0.00
Chromatiaceae	0.00	0.00	0.02	0.03	0.00	0.06	0.12	0.10	0.00	0.00	0.00	0.00	0.00	0.01	0.00	0.05
Streptosporangiaceae	0.00	0.00	0.00	0.02	0.00	0.00	0.00	0.00	0.00	0.00	0.00	0.00	0.00	0.00	0.00	0.00
Legionellaceae	0.00	0.00	0.00	0.02	0.00	0.06	0.01	0.00	0.00	0.00	0.00	0.00	0.00	0.00	0.00	0.00
Acidimicrobidae_incertae_sedis	0.00	0.00	0.00	0.02	0.00	0.00	0.00	0.00	0.00	0.00	0.04	0.00	0.00	0.00	0.00	0.00
Kineosporiaceae	0.00	0.00	0.00	0.02	0.00	0.02	0.00	0.07	0.00	0.00	0.00	0.00	0.00	0.01	0.00	0.00
Conexibacteraceae	0.00	0.00	0.00	0.01	0.00	0.00	0.00	0.00	0.00	0.00	0.00	0.00	0.00	0.00	0.00	0.00
Thermoleophilaceae	0.00	0.00	0.00	0.01	0.00	0.00	0.00	0.00	0.00	0.00	0.00	0.00	0.00	0.00	0.00	0.00
Nocardiopsaceae	0.00	0.00	0.00	0.01	0.00	0.00	0.00	0.00	0.00	0.00	0.00	0.04	0.00	0.00	0.00	0.00
Fusobacteriaceae	0.00	0.00	0.00	0.01	0.00	0.29	0.03	2.66	0.00	0.00	0.00	0.67	0.00	0.00	0.00	0.00
Actinosynnemataceae	0.00	0.00	0.00	0.01	0.00	0.07	0.00	0.00	0.00	0.00	0.00	0.00	0.00	0.00	0.00	0.00
Syntrophomonadaceae	0.00	0.00	0.00	0.01	0.00	0.00	0.00	0.00	0.00	0.00	0.00	0.00	0.00	0.00	0.00	0.00
Thermomonosporaceae	0.00	0.00	0.00	0.01	0.00	0.00	0.00	0.00	0.00	0.00	0.00	0.00	0.00	0.00	0.00	0.00
Patulibacteraceae	0.00	0.00	0.00	0.01	0.00	0.00	0.00	0.00	0.00	0.00	0.00	0.00	0.00	0.00	0.00	0.00
Bacteroidaceae	0.00	0.00	0.01	0.01	0.00	0.00	1.17	0.00	0.00	0.89	0.00	0.00	0.00	0.00	0.00	0.00
Gracilibacteraceae	0.00	0.00	0.00	0.01	0.00	0.00	0.00	0.00	0.00	0.00	0.00	0.00	0.00	0.00	0.00	0.00
Nannocystaceae	0.00	0.00	0.00	0.01	0.00	0.00	0.00	0.00	0.00	0.00	0.00	0.00	0.00	0.00	0.00	0.00
Polyangiaceae	0.00	0.00	0.00	0.01	0.00	0.00	0.00	0.00	0.00	0.00	0.00	0.01	0.00	0.00	0.00	0.00
Anaerolineaceae	0.00	0.00	0.00	0.01	0.00	0.00	0.00	0.00	0.02	0.00	0.00	0.00	0.00	0.00	0.00	0.00
Family XIII	0.00	0.00	0.00	0.01	0.00	0.02	0.00	0.00	0.00	0.00	0.00	0.00	0.00	0.00	0.00	0.00
Alphaproteobacteria_incertae_sedis	0.00	0.00	0.00	0.01	0.00	0.00	0.00	0.00	0.00	0.00	0.00	0.00	0.00	0.00	0.00	0.00
Sinobacteraceae	0.00	0.00	0.00	0.00	0.00	0.00	0.05	0.00	0.00	0.00	0.00	0.00	0.00	0.00	0.00	0.00
Alteromonadaceae	0.00	0.00	0.00	0.00	0.00	0.01	0.00	0.00	0.00	0.00	0.00	0.00	0.00	0.00	0.00	0.00
Pasteurellaceae	0.00	0.00	0.02	0.00	0.00	0.00	0.00	0.01	0.00	0.00	0.00	0.00	0.00	0.00	0.00	0.00
Aurantimonadaceae	0.00	0.00	0.00	0.00	0.00	0.01	0.00	0.00	0.00	0.00	0.00	0.47	0.00	0.00	0.00	0.02
Brevibacteriaceae	0.00	0.00	0.00	0.00	0.01	0.00	0.18	0.00	0.12	0.00	0.00	0.00	0.00	0.00	0.00	0.00
Beutenbergiaceae	0.00	0.00	0.00	0.00	0.00	0.01	0.00	0.00	0.00	0.00	0.00	0.00	0.00	0.00	0.00	0.00
Coriobacteriaceae	0.00	0.00	0.00	0.00	0.00	0.00	0.00	0.25	0.00	0.00	0.00	0.00	0.00	0.00	0.01	0.00
Actinomycetaceae	0.00	0.00	0.00	0.00	0.02	0.02	0.00	0.00	0.00	0.00	0.00	0.00	0.00	0.00	0.00	0.00
Bacillales_incertae_sedis	0.00	0.00	0.00	0.00	0.00	0.04	0.00	0.00	0.00	0.00	0.00	0.00	0.00	0.00	0.00	0.00
Caldilineaceae	0.00	0.00	0.01	0.00	0.00	0.00	0.00	0.00	0.03	0.00	0.00	0.00	0.00	0.00	0.00	0.00
Carnobacteriaceae	0.00	0.00	0.00	0.00	0.00	0.00	0.00	0.01	0.00	0.00	0.00	0.06	0.00	0.00	0.00	0.00
Coxiellaceae	0.00	0.00	0.00	0.00	0.00	0.00	0.00	0.00	0.00	0.00	0.00	0.00	0.00	0.00	0.00	0.00
Cystobacteraceae	0.00	0.00	0.00	0.00	0.00	0.00	0.01	0.00	0.00	0.00	0.00	0.00	0.00	0.00	0.00	0.00
Deinococcaceae	0.00	0.00	0.00	0.00	0.02	0.00	0.00	0.00	0.00	0.00	0.00	0.00	0.00	0.00	0.00	0.00
Dermabacteraceae	0.00	0.00	0.00	0.00	0.03	0.00	0.00	0.03	0.00	0.00	0.00	0.00	0.00	0.00	0.00	0.00
Desulfovibrionaceae	0.00	0.00	0.00	0.00	0.00	0.00	0.00	0.00	0.00	0.00	0.00	0.00	0.00	0.00	0.00	0.00
Erysipelotrichaceae	0.00	0.00	0.00	0.00	0.00	0.00	0.00	0.05	0.02	0.00	0.00	0.00	0.01	0.00	0.00	0.00
Halomonadaceae	0.00	0.00	0.00	0.00	0.00	0.00	0.00	0.00	0.00	0.00	0.00	1.18	0.00	0.00	0.00	0.83
Halothiobacillaceae	0.00	0.00	0.00	0.00	0.00	0.00	0.07	0.00	0.00	0.00	0.00	0.00	0.00	0.00	0.00	0.00

Helicobacteraceae	0.00	0.00	0.00	0.00	0.00	0.06	0.00	0.26	0.00	0.00	0.00	0.00	0.00	0.00	0.00	0.00
Incertae Sedis XII	0.00	0.00	0.00	0.00	0.00	0.00	0.00	0.03	0.00	0.00	0.00	0.00	0.00	0.00	0.00	0.00
Incertae Sedis XIV	0.00	0.00	0.00	0.00	0.00	0.00	0.25	0.00	0.00	0.00	0.00	0.00	0.00	0.00	0.00	0.00
Ktedonobacteraceae	0.00	0.00	0.00	0.00	0.00	0.00	0.21	0.00	0.00	0.00	0.00	0.10	0.00	0.00	0.00	0.00
Leptospiraceae	0.00	0.00	0.00	0.00	0.00	0.01	0.00	0.00	0.00	0.00	0.00	0.00	0.00	0.00	0.00	0.00
Leptotrichiaceae	0.00	0.00	0.00	0.00	0.00	0.00	0.02	0.00	0.06	0.00	0.00	0.00	0.00	0.00	0.00	0.00
Leuconostocaceae	0.00	0.00	0.00	0.00	0.00	0.01	0.00	0.00	0.00	0.00	0.00	0.00	0.00	0.00	0.00	0.00
Listeriaceae	0.00	0.00	0.00	0.00	0.00	0.00	0.00	0.80	0.00	0.00	0.00	0.00	0.00	0.00	0.00	0.00
Puniceicoccaceae	0.00	0.00	0.00	0.00	0.00	0.00	0.00	0.00	0.00	0.00	0.00	0.00	0.00	0.00	0.00	0.00
Rikenellaceae	0.00	0.00	0.00	0.00	0.00	0.00	0.01	0.00	0.00	0.00	0.00	0.00	0.00	0.00	0.00	0.00
Sporolactobacillaceae	0.00	0.00	0.02	0.00	0.00	0.00	0.00	0.00	0.00	0.00	0.00	0.00	0.00	0.00	0.00	0.00
Thermaceae	0.00	0.00	0.00	0.00	0.00	0.00	0.00	0.01	0.00	0.00	0.00	0.00	0.00	0.00	0.00	0.00
Thiotrichales_incertae_sedis	0.00	0.00	0.00	0.00	0.00	0.00	0.00	0.00	0.00	0.00	0.00	0.00	0.00	0.00	0.00	0.00
Xanthobacteraceae	0.00	0.00	0.02	0.00	0.02	0.00	0.00	0.00	0.00	0.03	0.00	0.08	0.00	0.00	0.00	0.00
Unclassified taxa	8.45	18.01	2.45	16.59	3.89	14.99	2.48	9.83	1.02	4.89	0.09	11.38	1.55	0.39	0.13	0.59

PBM, post blood meal
